# Short and Long-Term Outcomes in Kidney Transplant Recipients with Neutropenia or Leukopenia Following CMV Prophylaxis

**DOI:** 10.36469/001c.159410

**Published:** 2026-05-12

**Authors:** Vladimir Turzhitsky, Qinghua Li, Lei Ai, Murvin Jootun, Weijia Wang, Andrew Beyer, Pamela Moise, Irina Kolobova

**Affiliations:** 1 Merck & Co., Inc., Rahway, New Jersey; 2 Formerly of Merck & Co., Inc., Rahway, New Jersey; 3 Merck & Co., Inc., Rahway, NJ

**Keywords:** neutropenia, leukopenia, cytomegalovirus, healthcare cost, kidney transplant, healthcare resource utilization

## Abstract

**Background:**

Neutropenia and leukopenia following kidney transplant are linked to poor transplant outcomes. One factor that can contribute to neutropenia or leukopenia following transplant is the use of valganciclovir as a cytomegalovirus prophylaxis.

**Objective:**

This study aimed to evaluate post-transplant neutropenia and leukopenia and the associated resource use and costs among kidney transplant recipients receiving valganciclovir prophylaxis.

**Methods:**

Adults receiving kidney transplant with a claim for valganciclovir 30 days post-transplant were included (Merative™ MarketScan® with Medicare supplemental database, January 2012–March 2021). Incidence of, and risk factors for, neutropenia and leukopenia were assessed 1 year post-transplant. Clinical outcomes, resource use, and costs were assessed through 5 years post-transplant.

**Results:**

A total of 3121 and 357 individuals were included for the 1-year and 5-year analyses, respectively. Cumulative incidence of neutropenia or leukopenia 1 year post-transplant was 32.5%. Resource use was greater among patients with myelotoxicities than those without, driven by emergency department and inpatient visits. Among those with resource use, the total unadjusted median cost was greater in patients with vs without myelotoxicities (82 536vs68 652 per patient per year, respectively; *P* < .0001). A greater proportion of those with myelotoxicities experienced clinical complications and resource use 2 to 5 years post-transplant.

**Conclusions:**

Of kidney transplant recipients receiving valganciclovir prophylaxis, myelotoxicity claims were present in approximately one-third at 1 year post-transplant. Neutropenia or leukopenia in kidney transplant recipients is associated with high resource use and costs through 5 years post-transplant.

## INTRODUCTION

Cytomegalovirus (CMV) infection and disease can be associated with serious clinical complications after kidney transplant, including graft failure, graft rejection, and opportunistic infection.^1,2^ CMV-seronegative kidney transplant recipients (KTRs) (R−) with an allograft from a CMV-seropositive donor (D+) are at highest risk of post-transplant CMV infection and disease.^3^ At-risk KTRs are often administered CMV prophylaxis to minimize the risk of CMV infection/disease. In the United States (US), valganciclovir (or ganciclovir) prophylaxis has been the standard of care for CMV prevention among high-risk KTRs (D+/R−) since its approval by the US Food and Drug Administration (FDA) in 2003.^4,5^ In 2023, the FDA also approved letermovir prophylaxis for KTRs at high risk for CMV (D+/R−).^6^ Valganciclovir and letermovir have demonstrated effectiveness in reducing the risk of CMV infection and disease, as well as associated “indirect effects,” and are recommended in international consensus guidelines.^7–9^

Outcomes of kidney transplant can be impacted by the presence of neutropenia and/or leukopenia, which are frequently reported in KTRs.^10–13^ Valganciclovir has been associated with frequent neutropenia or leukopenia in randomized controlled trials, even at prophylaxis doses.^7,8^ Neutropenia has been reported in 13% to 48% of patients 1 year post-transplant^10^ and in 11% to 37% specifically following valganciclovir (or ganciclovir) administration.^12,14–16^ These myelotoxic side effects have further clinical consequences; KTRs who develop neutropenia or leukopenia following valganciclovir use are at an increased risk of graft rejection/failure, other opportunistic infections, and mortality.^11–13^ Notably, other etiologies of neutropenia and leukopenia in KTRs can contribute to poor clinical outcomes, including CMV serostatus and CMV disease, as well as other medications such as the immunosuppressant mycophenolic acid (MPA), in the form of mycophenolate mofetil or enteric-coated mycophenolate sodium, or antibacterials such as trimethoprim/sulfamethoxazole.^10,15,17^ In clinical practice, several approaches are possible to address the risk of post-transplant neutropenia and leukopenia, including reduction or discontinuation of MPA or trimethoprim/sulfamethoxazole and/or discontinuation or dose adjustment of valganciclovir.^10,13,17–19^ In high-risk KTRs, letermovir prophylaxis has similar efficacy against CMV outcomes vs valganciclovir but is associated with lower rates of neutropenia or leukopenia, therefore providing an alternative prophylactic option to reduce the risk of myelotoxicities.^7^ Conversion from valganciclovir to letermovir has also resulted in reduced neutropenia and reduced use of granulocyte colony-stimulating factor (G-CSF), which is used to counter myelotoxic side effects.^20^ Use of G-CSF can help manage neutropenia and leukopenia, however, manipulation of treatment and dosing can in itself result in CMV infection and graft rejection.^10,21^

Given the poor clinical outcomes, it is important to understand the economic burden associated with managing neutropenia and leukopenia in KTRs who receive CMV prophylaxis. It has been reported that neutropenia is associated with increased healthcare resource use (HCRU)^10^; however, the economic burden of neutropenia and leukopenia in KTRs receiving valganciclovir in the US has not yet been established and quantified using recent, large-scale data. Real-world data are therefore required to characterize the economic implications of post-transplant neutropenia and leukopenia.

This study aimed to evaluate the HCRU and cost of outcomes associated with post-transplant neutropenia and leukopenia after 1 year in KTRs receiving valganciclovir vs those without neutropenia and leukopenia. The long-term clinical and economic outcomes over 2 to 5 years post-transplant were also examined.

## METHODS

### Study Design

This was a US-based, retrospective, observational study of commercial claims data for KTRs at risk for CMV infection who were receiving valganciclovir prophylaxis. Data were obtained from the Merative™ MarketScan® Commercial and Medicare Databases (accessed August 2023), which includes de-identified patient-level data on age, gender, geographic region, HCRU, expenditures, and enrollment across inpatient, outpatient, prescription drug, and carve-out services. Costs are included from both commercial and Medicare claims. The index date was defined as the date of first kidney transplant with baseline data collected 1 year prior to the index date. Patients were followed up 1 year post-transplant to assess short-term outcomes and up to 5 years post-transplant (or until end of continuous enrollment) for long-term outcomes. All methods were performed in accordance with the relevant guidelines and regulations. It was not deemed necessary to obtain informed consent or ethics approval according to national regulations.

### Study Population

All individuals were aged ≥18 years (at the time of kidney transplant) with at least 1 procedure claim of kidney transplant between January 1, 2012, and March 31, 2021. Individuals with a history of kidney transplant, other solid organ transplant (including lung, liver, pancreas, bowel), or multiple organ transplants prior to the index date were not included. Individuals were required to have at least 1 year of continuous enrollment in medical and pharmacy benefits pre- and post-index or up to date of death (if death occurred within the first year post-index date).

All individuals were required to have received valganciclovir prophylaxis (filled ≥1 valganciclovir [450 mg/day or 900 mg/day] prescription within 30 days post-transplant, defined by National Drug Code). Valganciclovir use served as a proxy for patients at risk of CMV infection, as guidelines recommend valganciclovir for use in those at intermediate or high risk for CMV infection in the US.^5,9^

### Baseline and Follow-up Data Collection

Data on demographic, clinical, and transplant characteristics of patients were collected at baseline (1 year prior to kidney transplant) and reported for those with and without neutropenia or leukopenia.

All neutropenia and leukopenia events were identified as at least 1 claim with an *International Classification of Disease* (ICD) code (neutropenia: ICD-9 288.0x ICD-10 D70.x; leukopenia: ICD-9 288.5x, ICD-10 D72.81x (Table 1); the operational definition and code-list file is also provided in the **Supplementary Excel File**. The following outcomes were assessed at 1 year post-transplant: (1) cumulative incidence of neutropenia or leukopenia; time from kidney transplant to first neutropenia or leukopenia event and (2) the rate of neutropenia or leukopenia (per 100 patient-years). Neutropenia and leukopenia cohorts were defined using patient-years: for each patient, we identified any event occurring within a single follow-up year between 1 and 5 years post-transplant. We selected the follow-up year with continuous coverage and assigned the patient to the neutropenia or leukopenia cohort based on events in that year. All analyses were performed at the patient-year level.

**Table 1.** Clinical Outcome ICD Codes and Descriptions

**Outcome**	**ICD Code/Description**
Myelosuppression/toxicity	
Leukopenia	ICD-9 288.5x; ICD-10: D72.81x
Neutropenia	ICD-9 288.0x; ICD-10: D70.x
Thrombocytopenia	ICD-9 287.3–287.5; ICD-10: D69.4-D69.6
Opportunistic infection (other non-CMV infection)	
Bacterial infection	
Septicemia	ICD-9 038.x, 995.9x; ICD-10 A40.x, A41.x, A42.7
Pneumocystis pneumonia	ICD-9 136.3; ICD-10 B59
Tuberculosis (Mycobacterium tuberculosis)	ICD-9 010.x-018.x, ICD-10 A15.x-A19.x
Other bacterial	ICD-9 001.x-004.x, 008.0x-008.5x, 020.x-027.x, 030.x-036.x, 039.x-041.x, 073.x, 076.x, 080.x-83.x, 087.x-088.x, 091.x-100.x, 102.x, 104.x, 137.x; ICD-10 A00.x-A04.x, A20-A28, A30x-A39x, A42, A42.0-A42.2, A42.8-A42.9, A43.x-A49x, A70x, A71x, A75.x-A79.x, A68.x, A50.x-A64.x, A65.x-A69.x
Invasive fungal disease	
Candidiasis	ICD-9 112.4, 112.5, 112.81, 112.83, 112.84, 112.85, 112.89, 112.9x; ICD-10 B37.1, B37.7, B37.6, B37.84, B37.5, B37.81, B37.82, B37.89, B37.9
Coccidioidomycosis	ICD-9 114.0, 114.2, 114.3; ICD-10 B38.0, B38.4, B38.7x, B38.81
Blastomycosis	ICD-9 116.x; ICD-10 B40.0, B40.1, B40.2, B40.3, B40.7, B40.81, B40.89, B40.9, B41.0, B41.7, B41.8, B41.9, B48.0
Aspergillosis	ICD-9 117.3; ICD-10 B44.0, B44.7, B44.89, B44.1, B44.2, B48.4, B44.9
Cryptococcosis	ICD-9 117.5; ICD-10 B45.x
Histoplasmosis	ICD-9 115.01–115.05, 115.11–115.15, 115.91–115.95; ICD-10 B39.x
Other mycoses	ICD-9 117.6, 117.9; ICD-10 B48.2x, B48.3, B49
Zygomycosis	ICD-9 117.7; ICD-10 B46.x
Other/unspecified mycoses	ICD-9 117.9, 118.x; ICD-10 B48.x, B35.x, B36.x
Other viral infection	
Unspecified viral infections	ICD-9 079, 079.1-079.9x; ICD-10 B33.x, B34, B34.1-B34.9, B97.x, J20.3, J20.4, J20.5, J20.6, J20.7
Adenovirus	ICD-9 079.0; ICD-10 B34.0
BK virus	Billed as unspecified
Herpes simplex	ICD-9 054.9; ICD-10 B00.x, P35.2, A60.0x
Epstein-Barr virus	ICD-9 075.x; ICD-10 B27.0
Human herpes-6	ICD-9 058.21, 058.81; ICD-10 B00.1, B10.001, B10.81
Varicella zoster virus	ICD-9 052.9, 053.x; ICD-10 B01.x, B02.x, G02.0x, G05.1, J17.1
CMV infection/disease	ICD-9 078.5x; ICD-10 B25.0-9, B27.1x, H32.00, K87.00, K93.820
CMV infection	ICD-10 B25.9
csCMVi	ICD-10 B25.9
CMV disease	ICD-10 B25.0-8, B27.1x, H32.00, K87.00, K93.820
G-CSF use	
Injection, filgrastim (G-CSF), excludes biosimilars, 1 mcg	Code: J1442; Type: HCPCS
Injection, tbo-filgrastim, 1 mcg	Code: J1447; Type: HCPCS
Injection, plerixafor, 1 mg	Code: J2562; Type: HCPCS
Granulocytes, pheresis, each unit	Code: P9050; Type: HCPCS
Injection, filgrastim-sndz, biosimilar, (Zarxio), 1 mcg	Code: Q5101; Type: HCPCS
Injection, pegfilgrastim-jmdb, biosimilar, (Fulphila), 0.5 mg	Code: Q5108; Type: HCPCS
Injection, filgrastim-aafi, biosimilar, (Nivestym), 1 mcg	Code: Q5110; Type: HCPCS
Injection, pegfilgrastim-cbqv, biosimilar, (Udenyca), 0.5 mg	Code: Q5111; Type: HCPCS
Injection, pegfilgrastim-bmez, biosimilar, (ZIEXTENZO), 0.5 mg	Code: Q5120; Type: HCPCS
Home therapy; hematopoietic hormone injection therapy (eg, erythropoietin, G-CSF, GM-CSF); administrative services, professional pharmacy services, care coordination, and all necessary supplies and equipment (drugs and nursing visits coded separately)	Code: S9537; Type: HCPCS
Injection, pegfilgrastim, 6 mg	Code: J2505; Type: HCPCS
Injection, immune globulin (asceniv), 500 mg	Code: C9072, J1554; Type: HCPCS
Injection, immune globulin (bivigam), 500 mg	Code: J1556; Type: HCPCS
Injection, immune globulin (privigen), IV, non-lyophilized (eg, liquid), 500 mg	Code: J1459
Injection, immune globulin, (gammaplex), IV, non-lyophilized (eg, liquid), 500 mg	Code: J1557; Type: HCPCS
Injection, immune globulin, (gamunex-c/gammaked), non-lyophilized (eg, liquid), 500 mg	Code: J1561; Type: HCPCS
Injection, immune globulin, IV, lyophilized (eg, powder), not otherwise specified, 500 mg	Code: J1566; Type: HCPCS
Injection, immune globulin, (octagam), IV, non-lyophilized (eg, liquid), 500 mg	Code: J1568; Type: HCPCS
Injection, immune globulin, (gammagard liquid), non-lyophilized, (eg, liquid), 500 mg	Code: J1569; Type: HCPCS
Injection, immune globulin, (flebogamma/flebogamma dif), IV, non-lyophilized (eg, liquid), 500 mg	Code: J1572; Type: HCPCS
Injection, immune globulin, IV, non-lyophilized (eg, liquid), not otherwise specified, 500 mg	Code: J1599; Type: HCPCS
Injection, filgrastim (G-CSF), excludes biosimilars, 1 mcg	Code: J1442; Type: HCPCS
Graft failure	ICD-9 Dialysis Procedure Code after 35 days post KT or new transplant or ICD-9-PCS 55.53; ICD-10 T86.12 OR Dialysis Procedure Code after 35 days post KT or new transplant
Acute rejection	ICD-9 996.81 AND biopsy CPT code(s); ICD-10 T86.11

Abbreviations: CMV, cytomegalovirus; csCMVi, clinically significant cytomegalovirus infection; CPT, Current Procedural Terminology; G-CSF, granulocyte-colony stimulating factor; HCPCS, Healthcare Common Procedure Coding System; ICD, *International Classification of Diseases.*; KT, kidney transplant.

### HCRU and Costs

HCRU within 1 year of kidney transplant, including the baseline and follow-up periods, was quantified. HCRU outcomes included use of any inpatient or outpatient services, including the number of hospitalizations, emergency department (ED) visits, outpatient visits, office visits, and pharmacy claims.

All-cause total costs included cost of physician office visits, other outpatient and ED visits, and hospitalization and medication costs. For cost outcomes, total paid amount (plan-paid and patient-paid amount) was used as a proxy for cost. All cost data were adjusted to 2022 US dollars using the Consumer Price Index (CPI) from the Bureau of Labor Statistics for medical care and for prescription drugs.^22,23^ Analysis of costs for clinically significant CMV infection and CMV disease were restricted to year 2016 and after, as these clinical outcomes were only distinguishable in the ICD-10 coding system. Only patients with ≥98% completeness of nonzero cost data in the 1-year follow-up period were included.

### Long-term Clinical and Economic Outcomes

Clinical outcomes, HCRU, and the associated costs were also measured 2 to 5 years after kidney transplant (or end of continuous enrollment). Clinical outcomes included opportunistic infection, CMV infection, clinically significant CMV infection, CMV disease, G-CSF use, graft failure, acute rejection, new onset of diabetes mellitus after transplantation, and all-cause rehospitalization. To account for unknown ends of CMV episodes and to prevent potential double-counting, a 90-day washout period was implemented for CMV-related outcomes during event count (an event should not have any code indicating that specific outcome within the 90 days leading up to it). Costs associated with outcomes within 5 years were reported, including cost of medical services, inpatient hospitalization, ED visits, outpatient visits, office visits (as a subset of outpatient services), and pharmacy claims.

### Statistical Analyses

For all outcomes, analyses were conducted separately for cohorts of patients with “neutropenia *or* leukopenia,” patients with “neutropenia,” and patients with “leukopenia,” with results reported separately. The control cohorts consisted of those without a respective neutropenia or leukopenia event. The primary data reported here are for those with or without “neutropenia *or* leukopenia” with additional data for the individual “neutropenia” and “leukopenia” cohorts presented in the **Supplementary Material**.

All statistical analysis was conducted using SAS Studio 3.81 (SAS Institute Inc.). The predetermined statistical significance level was *P* < .05.

### Outcomes at 1-Year Post-transplant

The incidence of neutropenia or leukopenia was summarized as the number of patients who experienced an event during the first year of follow-up using raw count and percentage values. The rate of neutropenia or leukopenia was calculated as the total number of patients with events divided by the number of patient-years at risk and expressed as the number of events per 100 patient-years; 95% confidence intervals (CIs) for event rates were calculated using a Gaussian distribution. The cumulative incidence for the follow-up period was summarized using Kaplan-Meier curves. All incidence results were reported by duration of valganciclovir use in sensitivity analysis.

To determine factors associated with post-transplant neutropenia or leukopenia, a multivariable logistic regression model was used. A priori covariates of interest were medication use and characteristics, including antithymocyte globulin (ATG) use, alemtuzumab use, mycophenolate mofetil use, valganciclovir duration and dose, and trimethoprim-sulfamethoxazole use. Demographic characteristics and comorbidities were also included (see **Supplementary Table S1** for the complete list of candidate variables). The variables were selected through a combination of clinical input and an automated variable selection processes: Least Absolute Shrinkage and Selection Operator (LASSO). For the selected variables, the odds ratios from the logistic regression model were reported with 95% CIs. Unadjusted and adjusted models were estimated, and the goodness-of-fit was determined through C statistics.

The total and all-cause HCRU and costs were summarized using descriptive statistics (mean and median) per patient among those with resource use. The proportion use of resource among the entire cohort was summarized using percentage values. Characteristics were compared between cohorts with and without neutropenia and leukopenia using chi-square test for categorical variables and Wilcoxon rank sum tests for variables that did not have Gaussian distributions.

### 1-Year and 2- to 5-Year Analyses

In the 1-year and 2- to 5-year analyses, descriptive statistics were used to summarize baseline demographics and clinical characteristics for those with and without neutropenia or leukopenia, including mean, median, standard deviation (SD), and interquartile range. Characteristics were compared between cohorts using a *t*-test for continuous Gaussian variables, chi-square test for categorical variables, and Fisher’s exact test for categorical variables where there were fewer than 5 patients in any of the groups. Wilcoxon rank-sum tests were used for cost and count variables that do not have Gaussian distributions.

For the 1-year cost, multivariable linear regression models, with Charlson Comorbidity Index scores and duration of valganciclovir use as covariates, were run for each outcome of interest and for the cohort with and without neutropenia or leukopenia separately. The least-square marginal means were then used to estimate the cost for patients with and without the outcome of interest. Cost per event was calculated by dividing the total cost for the outcome by the average number of events.

From the clinical perspective, it is hypothesized that, beyond 1 year, KTRs are healthier and there would not be an increase in cost per event between the leukopenia/neutropenia and the no leukopenia/neutropenia cohort. Additionally, as the sample size was expected to be limited after 2 years, it was not deemed feasible to conduct the analysis with the cohort separated. Hence, the cohorts were combined to increase the power of the analysis to obtain the cost per event regardless of leukopenia/neutropenia status.

Healthcare costs associated with outcomes of interest were calculated for years 2 to 5 post-transplant. Costs associated with each outcome of interest were summarized for the cohorts with and without neutropenia or leukopenia, with the number of events reported for each outcome. Adjusted costs associated with each outcome of interest were estimated using multivariable linear regression models.

## RESULTS

### Study Population

**Figure 1.**
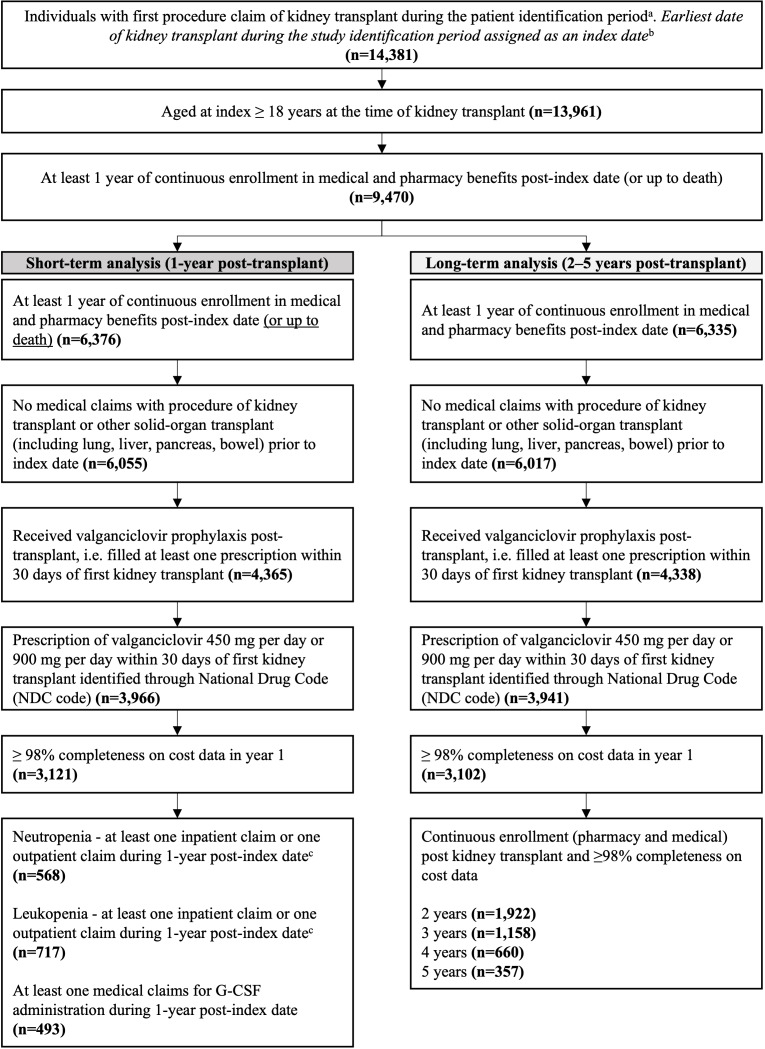
Participant Flow Abbreviations: G-CSF, granulocyte-colony stimulating factor; KT, kidney transplant; NDC, National Drug Code; SOT, solid-organ transplant ^a^Patient identification period: January 1, 2012–March 31, 2021. ^b^Index date: Date of first kidney transplant during the patient identification period. ^c^1013 patients had either neutropenia or leukopenia claims. 272 patients had both neutropenia and leukopenia claims.

A total of 14 391 individuals had received a first kidney transplant between January 1, 2012, and March 31, 2021 (Figure 1). Of these, 3121 individuals were eligible for the 1-year analysis and 3102 for the 2- to 5-year analysis (Figure 1).

### Outcomes at 1 Year Follow-up

**Figure 2.**
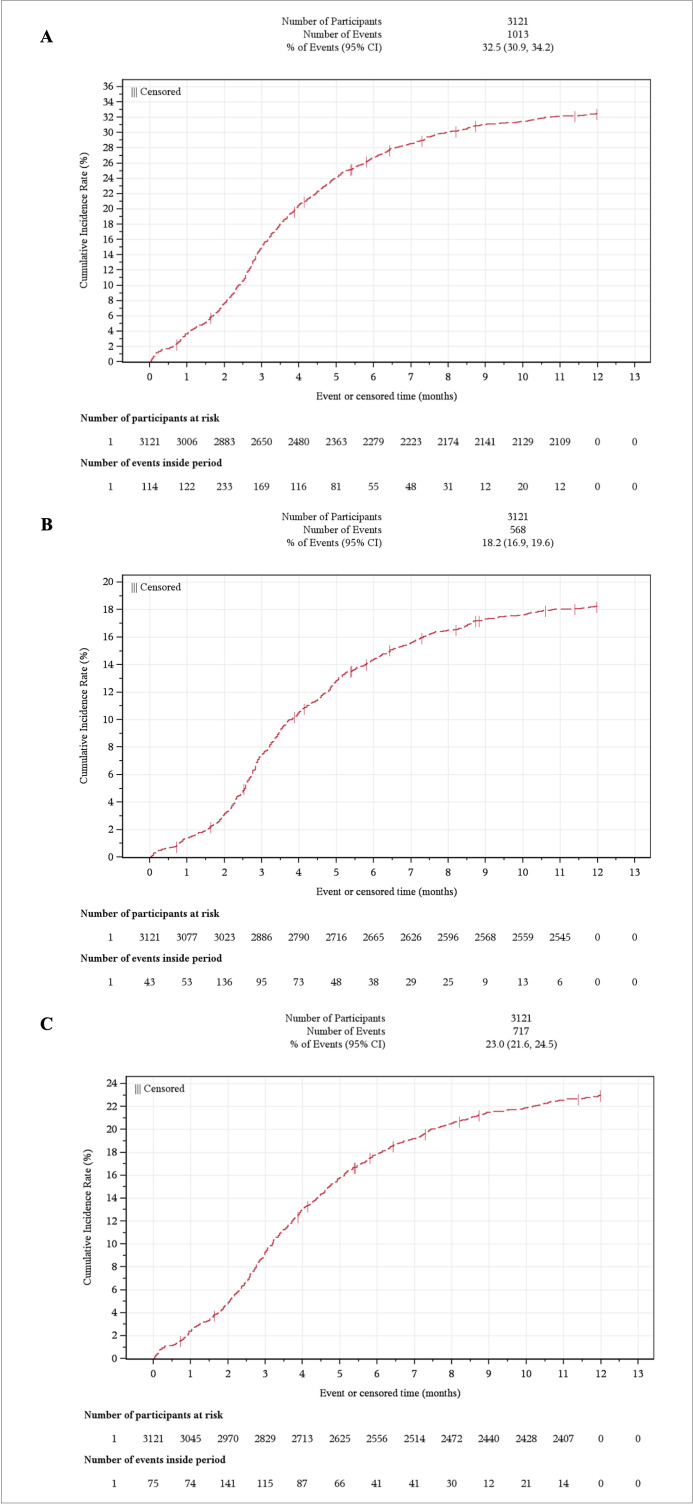
Kaplan-Meier Curve for the Cumulative Incidence of Neutropenia or Leukopenia: (**A**) Neutropenia Only, (**B**) Leukopenia Only, (**C**) 1 Year Post-transplant Abbreviation: CI, confidence interval.

The cumulative incidence of either neutropenia or leukopenia claims during the first year post-transplant was 32.5% (95% CI, 30.9-34.2) (Figure 2A). A total of 18.2% (95% CI, 16.9-19.6) developed neutropenia (Figure 2B), with an incidence rate of 20.7% (95% CI, 19.1-22.5) per 100 patient-years. Meanwhile, 23.0% (95% CI, 21.6-24.5) developed leukopenia (Figure 2C) at a rate of 27.1% (95% CI, 25.1-29.2) per 100 patient-years. The mean (SD) time after transplant was 125 (76.1) days for the first neutropenia event and 127 (83) days to the first leukopenia event.

### Valganciclovir Use with and without Neutropenia or Leukopenia

Across cohorts with and without neutropenia or leukopenia, most patients received valganciclovir prophylaxis for less than 100 days after kidney transplant (56.8%), with only 10.1% receiving valganciclovir prophylaxis beyond 200 days post-transplant. Though duration of valganciclovir use was not significantly different between those with and without neutropenia or leukopenia, this result cannot be inferred to have any causal relationship, as the onset of neutropenia or leukopenia would result in a shortened duration of valganciclovir use. A different study design would be required to evaluate their causal relationship. A total of 44.4% with neutropenia or leukopenia discontinued valganciclovir for at least 15 days (Table 2), a significantly greater proportion vs the 31.5% discontinuation rate for those without neutropenia or leukopenia (*P* < .0001).

**Table 2.** Baseline^a^ Characteristics and Valganciclovir Use Up to 1 Year Post-transplant for Those with and without Neutropenia or Leukopenia

**Characteristics**	**With Neutropenia or Leukopenia, n = 1013**	**Without Neutropenia or Leukopenia, n = 2108**
Age, years		
Median (P25, P75)**	52 (43, 60)	54 (45, 61)
≥65	103 (10.2)	259 (12.3)
Gender, n (%)		
Male	604 (59.6)	1254 (59.5)
Geographic region: OPTN^b,^**, n (%)		
Region 1 (Northeast)	24 (2.4)	68 (3.2)
Region 2 (South, Northeast)	71 (7.0)	184 (8.7)
Region 3 (South)	206 (20.3)	335 (15.9)
Region 4 (South)	93 (9.2)	188 (8.9)
Region 5 (West)	94 (9.3)	229 (10.9)
Region 6 (West)	29 (2.9)	60 (2.8)
Region 7 (Midwest)	51 (5.0)	127 (6.0)
Region 8 (Midwest, West)	37 (3.7)	95 (4.5)
Region 9 (Northeast)	90 (8.9)	229 (10.9)
Region 10 (Midwest)	110 (10.9)	263 (12.5)
Region 11 (South)	189 (18.7)	305 (14.5)
Unknown	19 (1.9)	25 (1.2)
Primary payer type*, n (%)		
Commercial	893 (88.2)	1794 (85.1)
Medicare	120 (11.8)	314 (14.9)
Charlson Comorbidity Index^c^, n (%)		
0	327 (32.3)	682 (32.4)
1-2	310 (30.6)	631 (29.9)
3-4	268 (26.5)	536 (25.4)
≥5	108 (10.7)	259 (12.3)
Calendar year of transplant**, n (%)		
2012	81 (8.0)	335 (15.9)
2013	75 (7.4)	263 (12.5)
2014	99 (9.8)	240 (11.4)
2015	104 (10.3)	267 (12.7)
2016	139 (13.7)	227 (10.8)
2017	130 (12.8)	227 (10.8)
2018	103 (10.2)	151 (7.2)
2019	141 (13.9)	192 (9.1)
2020	111 (11.0)	166 (7.9)
2021	30 (3.0)	40 (1.9)
Comorbidities^d^, n (%)		
Diabetes and diabetes with complications**	527 (52.0)	1226 (58.2)
Congestive heart disease	154 (15.2)	369 (17.5)
Mild and moderate liver disease	140 (13.8)	276 (13.1)
Chronic pulmonary disease	111 (11.0)	229 (10.9)
Cancer	65 (6.4)	165 (7.8)
Rheumatological disease*	65 (6.4)	94 (4.5)
Induction therapy^e^, n (%)		
Antithymocyte globulin* n (%)	57 (5.6)	83 (3.9)
Basiliximab	23 (2.3)	39 (1.9)
Maintenance therapy^e^, n (%)		
Tacrolimus**	702 (69.3%)	1359 (64.5%)
Mycophenolate mofetil	558 (55.1%)	1187 (56.3%)
Prednisone*	681 (67.2%)	1492 (70.8%)
Methylprednisolone**	142 (14.0%)	216 (10.2%)
VGCV use post-transplant:^h^ Initiation and discontinuation		
Duration of VGCV use (calculated before discontinuation), n (%)		
<100 days	569 (56.2)	1204 (57.1)
100-199 days	353 (34.8)	681 (32.3)
≥200 days	91 (9.0)	223 (10.6)
Days from index to first VGCV fill 450 mg/day dosage		
Median (P25, P75)	3 (1, 5)	3 (1, 4)
Days from index to first VGCV fill 900 mg/day dosage		
Median (P25, P75)	2 (1, 4)	3 (1, 5)
Discontinued VGCV with a gap ≥15 days***, n (%)		
Yes	450 (44.4)	664 (31.5)

Abbreviations: CDHP, consumer directed health plan; CI, confidence interval; EPO, exclusive provider organization; HCPCS, Healthcare Common Procedure Coding System; HDHP, high-deductible health plan; HMG-CoA, 3-hydroxy-3-methylglutaryl coenzyme A; HMO, health maintenance organization; NDC, National Drug Code; OPTN, Organ Procurement and Transplantation Network; P, percentile: POS, point of service; PPO, preferred provider organization; SD, standard deviation; VGCV, valganciclovir.

^a^ One-year prior to kidney transplant.

^b^ Unknown region: No state-level data available, or Vermont.

^c^ Excludes renal disease.

^d^ Comorbidities with a prevalence <1% not reported.

^e^ Include baseline period, index hospitalization and up to 14 days after kidney transplant period.

^f^ NDC and HCPCS codes were not present in baseline period, index hospitalization and up to 14 days post-kidney transplant period.

^g^ NDC and HCPCS codes were unavailable.

^h^ Data relate to 1 year after kidney transplant.

*P < .05.

**P < .01.

***P < .0001.

### Patient Characteristics

Baseline demographic, clinical, and transplant characteristics for those with (n = 1013) and without neutropenia or leukopenia (n = 2108) were largely comparable (Table 2). Patients with neutropenia or leukopenia were younger than those without (median 52 vs 54 years, *P* < .001). A higher proportion of those with neutropenia or leukopenia had rheumatological disease (6.4% vs 4.5%; *P* = .0235). Diabetes and metastatic cancer, however, were more common among those without neutropenia or leukopenia (*P* = .0001 and *P* = .0068, respectively).

A higher proportion of those with vs without neutropenia or leukopenia received tacrolimus (69.3% vs 64.5%; *P* = .008) and ATG (5.6% vs 3.9%; *P* = .03).

Across both groups, a steady decline in the number of KTRs was observed over time due to the decrease in population coverage of the MarketScan® database, with 416 transplants performed in 2012 vs 70 in 2021. There was a significant association between calendar year of transplant and myelosuppression events (neutropenia or leukopenia), with a higher proportion of events in more recent years (chi-square test, *P* < .001). Further, higher proportions of KTRs with vs without neutropenia or leukopenia received their transplant in recent years (after 2016).

**Supplementary Table S2** reports baseline data for myelosuppression subgroups.

### Factors Associated With Neutropenia or Leukopenia

Variables selected by the LASSO for inclusion in the logistic regression analysis are presented in **Supplementary Table S3**. The logistic regression had a C statistic of 0.635. Those receiving a transplant in 2016 or later experienced significantly greater odds of developing post-transplant neutropenia or leukopenia relative to those receiving a transplant in 2012 (*P* < .0001). In relation to comorbidities, rheumatology was associated with increased odds of developing post-transplant neutropenia or leukopenia (OR, 1.481 [95% CI, 1.049, 2.09]; *P* = .0256). Certain induction and maintenance therapies and geographical regions (Organ Procurement and Transplantation Network [OPTN]) were also found to be associated with the risk of post-transplant neutropenia or leukopenia.

### Baseline HCRU and Costs with and without Neutropenia and Leukopenia

HCRU and costs at baseline (1 year before kidney transplant) were generally comparable between groups. However, a significantly greater proportion of patients who went on to have a neutropenia or leukopenia event during the first year post-transplant had a toxicity-related ED visit during the baseline period than those who did not develop neutropenia or leukopenia.

### Economic Outcomes at 1 Year

Among the entire cohort, 100% of KTRs had recorded all-cause outpatient visits and pharmacy use 1 year post-transplant, regardless of neutropenia or leukopenia status (**Supplementary Table S4**). However, a greater proportion of KTRs with neutropenia or leukopenia visited the ED (61% vs 53%) or used inpatient services (51% vs 38%) compared with those without. Similar outcomes were recorded among neutropenia (**Supplementary Table S5**) and leukopenia (**Supplementary Table S6**) subgroups.

Among KTRs with resource use, unadjusted HCRU (ED visits, other outpatient visits, and hospitalizations) and costs were greater among those with vs without neutropenia or leukopenia (**Supplementary Table S4** and **Supplementary Figure S1A**). Among those with a claim for an outpatient visit (other than ED), inpatient hospitalization, or CMV-related prescription, the mean utilization per patient was significantly higher in those with vs without neutropenia or leukopenia (*P* < .0001 for all). Similar results were observed for neutropenia and leukopenia independently.

The total unadjusted annual median cost per patient among those with resource use was significantly greater for those with neutropenia or leukopenia than those without ($82 536 vs $68 652; *P* < .0001; **Supplementary Figure S1A**). Unadjusted outpatient (other than ED) and inpatient visit costs were significantly greater in those with vs without neutropenia or leukopenia (*P* = .0003 and *P* < .0001, respectively; **Supplementary Figure S1A**). Similar findings were observed for neutropenia (**Supplementary Figure S1B**) and leukopenia subgroups (**Supplementary Figure S1C**). Unadjusted HCRU and costs at 1-year follow-up for neutropenia and leukopenia are shown in **Supplementary Table S5** and **Supplementary Table S6**, respectively.

In the adjusted analysis, patients without a complete year of follow-up, including those who died, were excluded to ensure accurate estimation of annual post-transplant costs. Those with post-transplant neutropenia or leukopenia had higher costs per clinical event at 1-year follow-up vs those without, except for G-CSF use (Table 3). In both those with and without neutropenia or leukopenia, CMV disease was associated with a higher adjusted cost per event compared with clinically significant CMV infection and CMV infection (Table 3). Data for neutropenia and leukopenia alone are presented in **Supplementary Table S7**.

**Table 3.** Differences in Adjusted Costs Between Those with and without Neutropenia or Leukopenia, Neutropenia Alone, and Leukopenia Alone, 1 Year Post-Transplant, from Multivariable Models for Clinical Outcomes of Interest^a^

**Event**	**With Neutropenia or Leukopenia, n=1013**				**Without Neutropenia or Leukopenia, n=2108**				**Difference Between Cohorts, $**
**Difference (with and without Events), $**	**Low-High Confidence Level, $**	**No. of Events**	**Cost Difference per Event, $**	**Difference (with and without Events), $**	**Low-High Confidence Level, $**	**No. of Events**	**Cost Difference per Event, $**		
CMV infection	18 032	15 339-21 197	1.36	13 218	12 118	10 203-14 392	1.37	8827	5914
CsCMVi	40 993	33 762-49 773	1.03	39 698	23 792	18 441-30 697	1.04	22 859	17 201
CMV disease	55 863	43 510-71 722	1.21^b^	55 863	42 264	28 335-63 040	1.00	42 264	13 599
Acute graft rejection	57 221	50 904-64 321	1.33	43 109	34 465	31 959-37 168	1.28	26 968	22 756
Graft failure	50 071	42 889-58 455	1.00	50 071	29 067	25 758-32 802	1.00	29 067	21 004
Viral, fungal, or bacterial opportunistic infection	30 661	27 783-33 837	5.73	5 355	26 593	24 831-28 480	4.83	5503	4068
G-CSF	21 099	18 993-23 438	3.10	6805	53 764	47 440-60 931	3.20	16 808	−32 665
Rehospitalization	75 915	69 554-82 858	1.86	40 859	66 202	62 342-70 301	1.53	43 292	9713
Other	−50 683	–	–	–	−40 480	–	–	–	−10 203

Abbreviations: CMV, cytomegalovirus; csCMVi, clinically significant cytomegalovirus infection; G-CSF, granulocyte-colony stimulating factor.

^a^Analysis excludes patients who died prior to 1 year post-transplant (ie, died mid-year).

^b^Clinical experts have noted that the CMV disease event rate observed in clinical practice is higher. This elevated rate observed in the database analysis could be attributed to multiple inputs and the misclassification of ICD codes related to CMV. Consequently, it is assumed that the event rate would be 1, signifying 1 CMV disease event per patient per year.

### Outcomes at 2- to 5-Year Follow-up

**Clinical outcomes:** In the 2- to 5-year follow-up post-transplant, a greater proportion of patients with vs without post-transplant neutropenia or leukopenia experienced CMV infection, clinically significant CMV infection, CMV disease, and other opportunistic infections, as well as poor transplant outcomes, such as graft failure and acute rejection, and all-cause rehospitalization (**Supplementary Table S8**). G-CSF use was also higher among those with vs without post-transplant neutropenia or leukopenia (**Supplementary Table S8**).

**Economic outcomes:** In the unadjusted analysis for all patients, higher overall mean costs 2 to 5 years post-transplant were found among those with neutropenia or leukopenia vs those without ($80 237 vs $50 128) (**Supplementary Table S8**). This was consistent for costs associated with all clinical outcomes investigated except G-CSF use, where mean costs were higher for those without neutropenia or leukopenia (**Supplementary Table S8**). The total unadjusted annual median cost per patient among those with resource use was significantly greater for those with neutropenia or leukopenia than those without ($26 389 vs $20 052; *P* < .0001) (**Supplementary Figure S2A**). Similar results were observed for those with neutropenia only (**Supplementary Figure S2B**) and leukopenia only (**Supplementary Figure S2C**).

In the adjusted analysis for years 2 to 5 post-transplant, there was no significant difference in cost between groups, so data were pooled for those with and without neutropenia or leukopenia. In the combined cohort of KTRs receiving valganciclovir either with or without neutropenia or leukopenia, CMV disease ($141 087), clinically significant CMV infection ($59 987), and acute rejection ($33 556) were the clinical outcomes with the highest adjusted cost difference per event (Table 4).

**Table 4.** Differences in Adjusted Costs Between Those with and without Clinical Outcome Event 2-5 Years Post-Transplant from Multivariable Models for Clinical Outcomes of Interest^a^

**Clinical Outcome Event**	**Difference (with and without Event), $**	**High-Low Confidence Level, $**	**No. of Events**	**Cost Difference per Event, $**
CMV infection	13 675	7603-24 596	1.69	8098
CsCMVi	67 044	15 984-281 206	1.12	59 987
CMV disease	176 359	164 666-188 882	1.25	141 087
Acute graft rejection	41 556	36 867-46 842	1.24	33 556
Graft failure	26 131	23 692-28 822	1.00	26 131
Viral, fungal, or bacterial opportunistic infection	16 691	14 221-19 590	3.43	4865
G-CSF	70 385	57 812-85 692	3.81	18 495
Rehospitalization	59 974	55 913-64 330	2.81	21 374

Abbreviations: CMV, cytomegalovirus; csCMVi, clinically significant cytomegalovirus infection; G-CSF, granulocyte-colony stimulating factor.

^a^Analysis excludes patients who died prior to 1 year post-transplant (ie, died mid-year).

## DISCUSSION

This study aimed to quantify the burden of neutropenia and leukopenia after kidney transplant in a population of patients receiving valganciclovir CMV prophylaxis and assess associated HCRU and cost. Approximately one-third of KTRs who received valganciclovir developed either neutropenia or leukopenia in the 1 year post-transplant. Over 1-year follow-up, all-cause ED visits, outpatient visits and hospitalizations, and the associated costs were higher among those with vs without neutropenia or leukopenia. In long-term analyses, those with neutropenia or leukopenia experienced worse clinical outcomes 2 to 5 years post-transplant, including increased opportunistic infections and CMV infection/disease, poorer graft outcomes and higher rates of rehospitalization, leading to increased HCRU-related costs up to 5 years post-transplant.

While the current study reported a cumulative incidence of 32.5% 1 year post-transplant in KTRs receiving valganciclovir, others have previously reported that as many as 89.6% of KTRs receiving valganciclovir prophylaxis develop leukopenia, 57.2% develop moderate neutropenia, and 11.3% develop severe neutropenia.^24^ Differences may be explained by variations in the operational definition used to classify events; this study used administrative billing claims diagnosis codes for identifying neutropenia and leukopenia, which tend to underrepresent cases from true blood laboratory values. Consequently, this study considered claims for neutropenia *or* leukopenia with the aim to better assess the true burden of myelotoxicity post-transplant. Overall, the current study reiterated the burden of post-transplant neutropenia or leukopenia among KTRs receiving valganciclovir prophylaxis. It is important to recognize, however, that this study did not investigate causality between medication use and neutropenia or leukopenia. Among KTRs, various etiologies of neutropenia and leukopenia can contribute to poor clinical outcomes, including CMV serostatus and CMV disease in addition to use of medications other than valganciclovir such as MPA and trimethoprim/sulfamethoxazole.^10,15,17^ The multivariate model for the risk of neutropenia/leukopenia used in this study accounted for medications as covariates.

Neutropenia and leukopenia can lead to further complications post-transplant, as seen in this study. A 2023 systematic literature review of 82 observational studies reported a positive association between neutropenia and subsequent opportunistic infection and graft rejection in KTRs.^10^ A separate US retrospective cohort study of high-risk (D+/R−) KTRs (n = 572; 2012-2018) who received 6 months valganciclovir prophylaxis following transplant found that the most common complications associated with post-transplant neutropenia were first-graft rejection (53.4%) and CMV viremia (24.5%).^24^ While the relationship between neutropenia and leukopenia and subsequent graft failure or mortality after kidney transplant is reported less consistently,^10^ a US observational study reported KTRs who developed neutropenia following valganciclovir or ganciclovir use had significantly higher rates of graft rejection, graft failure, and G-CSF use than those without neutropenia (all *P* < .001), with a similar pattern observed in the case of those with leukopenia.^11^ Similarly, a large-scale US database study with an 84-month follow-up reported increased risk of both graft failure and mortality.^15^ The increased rate of opportunistic infections, poor transplant outcomes, and rehospitalization in the 2 to 5 years post-transplant observed in our study provides further evidence of the broad negative impact of neutropenia and leukopenia on post-transplant clinical outcomes.

Moreover, this study highlighted the economic burden associated with poor clinical outcomes among KTRs, both 1 year and 2 to 5 years post-transplant, consistent with current literature. Previous studies reported increased HCRU among those with vs without neutropenia,^10,25^ with one study reporting the risk of hospitalization to be 3-times higher among those with neutropenia and similar trends for leukopenia.^25^ Raval et al similarly reported significantly higher rehospitalization among those with neutropenia (61.5% vs 55.3%) and leukopenia (69.7% vs 30.3%) post-transplant compared with those without (*P* < .001 for both).^26^ Brar et al also found that patients with neutropenia have a higher incidence rate of first hospitalization and shorter time to hospitalization vs those without.^18^ Increased HCRU subsequently drives higher costs in this population. Previous analyses have shown that increased 1-year HCRU among patients with vs without post-transplant neutropenia or leukopenia resulted in increased costs per patient of $22 911.^25^ This compares to the findings of this study, where high HCRU among those with neutropenia or leukopenia led to a median 1-year total unadjusted cost that was $13 884 higher per patient relative to those without neutropenia or leukopenia. The higher costs previously reported can be explained by the requirement for two diagnosis codes ≥14 days apart, indicative of a more persistent condition,^25^ whereas the current study only required a single claim. The higher adjusted costs observed for G-CSF use among patients without neutropenia or leukopenia were unexpected and merit further investigation. One possible explanation is that G-CSF use in this group may identify patients with greater illness severity and higher associated healthcare costs.

Overall, this study provides further evidence that myelotoxicities post-transplant are present among KTRs receiving valganciclovir and can generate a clinical and economic burden for this population. Hesitation around valganciclovir administration among healthcare professionals has been reported due to the associated risk of myelotoxicities.^27^ While not investigated in the current study, alternative CMV prophylactic agents without the association of myelotoxic side effects could contribute to reducing the clinical and economic burden post–kidney transplant. At the time of this study, there were no other approved CMV prophylactic agents for use in KTRs, limiting the opportunity to make direct comparisons. Since then, however, letermovir, an alternative CMV prophylactic agent, received FDA approval for use in high-risk adult KTRs in 2023.^6^ Unlike valganciclovir, clinical trials suggest that letermovir use is not associated with myelosuppressive side effects, thus may contribute to reduced economic burden post–kidney transplant.^7^ Once real-world evidence for letermovir use accumulates, there will be opportunity to evaluate the incidence of neutropenia and leukopenia in KTRs to add to the findings presented here.

### Limitations

While real-world data can provide valuable insights to complement traditional experimental data, any interpretations should consider all associated limitations, such as the impact of unmeasured, confounding, and missing data.^28,29^

This study reported the burden of neutropenia and/or leukopenia in KTRs who received valganciclovir; however, this study did not include a comparator group of KTRs who did not receive valganciclovir, and therefore the direct association between valganciclovir and neutropenia or leukopenia was not assessed. Neutropenia and leukopenia have several etiologies in KTRs, and the neutropenia/leukopenia in patients included in this analysis may not be the result of valganciclovir. Further analysis would be needed to explore this association.

This study aimed to quantify all-cause utilization and costs, accounting for patient characteristics, rather than limiting analysis to kidney transplant-related outcomes only. While the HCRU and costs presented here may not be associated with kidney transplant and related sequelae, considering the variation in coding practices across the US, the methodology applied here provided a comprehensive approach to capture the costs of care that may have been induced or exacerbated by kidney transplant. Moreover, clinical outcomes of interest included CMV and other viral, fungal and bacterial infections, which may not have been captured with kidney transplant as the primary diagnosis code, therefore considering all-cause costs rather than kidney-transplant-related costs allowed for analysis of these outcomes.

The Merative™ MarketScan® database covers individuals insured through large US employers, as well as those with supplemental Medicare coverage; therefore, the study population may not truly reflect the real-world US kidney transplant population, where the most frequent age bracket is >50 years.^30^ While the observed reduction in transplant procedures in recent years observed in this study may be explained by Merative™ MarketScan® having a reduced population pool over time,^30^ further studies are required to fully characterize the rate of kidney transplant and burden of CMV-related complications in the US population over 50 years of age.

Further, CMV serostatus information is not available through the Merative™ MarketScan® database. To address this, the study sample was restricted to those receiving valganciclovir, as valganciclovir is only indicated for high-risk populations (D+/R−) in the US.^5^ However, current guidelines recommend valganciclovir use among patients with intermediate risk (R+)^9^; therefore, the study population could include a mixed population of patients with high or intermediate risk.

In addition, due to the limitation of ICD codes, clinically significant CMV infection and CMV disease are not distinguishable in the ICD-9 era and only distinguishable in the ICD-10 era. Therefore, adjusted costs from those outcomes were only from data at and after 2016. The identification of neutropenia and leukopenia solely relying on ICD codes may only capture more severe and persistent cases. The lack of specific lab test data limits the ability to accurately capture all instances of neutropenia and leukopenia. In the claims database, neutropenia events may be listed as neutropenia or leukopenia; therefore, this combined grouping was considered the key data subset in this study to provide a more realistic representation of neutropenia after kidney transplant. Some patients were reported to not receive induction or maintenance therapy which may relate to the definitions used to identify the study population within the database. While this study may represent an underestimate of the total population of KTRs with neutropenia or leukopenia, this is not expected to impact its conclusions. A previous study using lab-based neutropenia/leukopenia criteria showed consistent trends with the claims-based definition used here in relation to HCRU.^31^ Future work might focus on determining the accuracy of the surrogate claims-based definition for neutropenia/leukopenia.

Since the national death index is not available through the Merative™ MarketScan® database, mortality was identified only through evidence of discharge outcome of death, meaning that mortality may be underestimated. Additionally, due to privacy concerns, the death discharge status was classified as “missing” post-2016. Therefore, this study is not well positioned to make conclusions on mortality.

Finally, the low patient number for some clinical outcomes within the 2- to 5-year analysis risks the related study outputs lacking generalizability for the wider population.

## CONCLUSION

This US database study demonstrates that neutropenia or leukopenia is present in approximately one-third of KTRs receiving valganciclovir prophylaxis. The presence of neutropenia or leukopenia post-kidney transplant drives increased HCRU, resulting in higher costs in the 1 year post-transplant vs those without neutropenia or leukopenia, with the economic burden persisting up to 5 years post-transplant. This supports previous findings that post-transplant myelotoxicities in KTRs generate increased clinical and economic burden in the US, highlighting the need to address the risk of post-transplant neutropenia and leukopenia. While not investigated here, the use of alternative FDA-approved CMV prophylactic agents that are not associated with neutropenia and leukopenia may reduce the burden of disease. As such, future research should re-evaluate the real-world burden of neutropenia and leukopenia in KTRs receiving CMV prophylactic agents that have since become available.

### Ethics Approval and Consent to Participate

As this study involved analysis of pre-existing, deidentified data (that is not re-identifiable), it did not require institutional review board (IRB) review or a waiver per Federal Regulations for the protection of Human Research Subjects (45 CFR §46), and patient consent was not required.

### Disclosures

At the time of the study, all authors were employees of Merck Sharp & Dohme LLC, a subsidiary of Merck & Co., Inc., Rahway, New Jersey, USA, which funded this study. All authors declare no other conflicts of interest.

## Supplementary Material

Online Supplementary Material

Supplementary Excel File

## Data Availability

The data that support the findings of this study are available from the Merative^™^ MarketScan^®^, but restrictions apply to the availability of these data, which were used under license for the current study, and so are not publicly available. The data are commercially available from Merative^™^ Marketscan^®^ in the US upon license; the authors cannot provide the data upon request. Please contact the corresponding author with questions about the data.

## References

[1] Raval A. D., Kistler K. D., Tang Y., Murata Y., Snydman D. R. (2021). Epidemiology, risk factors, and outcomes associated with cytomegalovirus in adult kidney transplant recipients: a systematic literature review of real‐world evidence. Transpl Infect Dis.

[2] Jorgenson M. R., Descourouez J. L., Cardinale B.. (2019). Risk of opportunistic infection in kidney transplant recipients with cytomegalovirus infection and associated outcomes. Transpl Infect Dis.

[3] Sagedal S., Nordal K. P., Hartmann A.. (2000). A prospective study of the natural course of cytomegalovirus infection and disease in renal allograft recipients. Transplantation.

[4] Raval A. D., Ganz M. L., Fraeman K.. (2022). Real-world treatment patterns of antiviral prophylaxis for cytomegalovirus among adult kidney transplant recipients: a linked USRDS-Medicare database study. Transplant Int.

[5] US Food and Drug Administration Valganciclovir prescribing information.

[6] US Food and Drug Administration Letermovir prescribing information.

[7] Limaye A.P., Budde K., Humar A.. (2023). Letermovir vs valganciclovir for prophylaxis of cytomegalovirus in high-risk kidney transplant recipients: a randomized clinical trial. JAMA.

[8] Humar A., Lebranchu Y., Vincenti F.. (2010). The efficacy and safety of 200 days valganciclovir cytomegalovirus prophylaxis in high-risk kidney transplant recipients. Am J Transplant.

[9] Kotton C. N., Kumar D., Manuel O.. (2025). The Fourth International Consensus Guidelines on the management of cytomegalovirus in solid organ transplantation. Transplantation.

[10] Raval A. D., Kistler K. D., Tang Y., Vincenti F. (2023). Burden of neutropenia and leukopenia among adult kidney transplant recipients: a systematic literature review of observational studies. Transpl Infect Dis.

[11] Raval A. D., Turzhitsky V., Fazio-Eynullayeva E., Jin H., Merchant S. (2022). 207. Burden of post-transplant neutropenia and leukopenia among kidney transplant recipients: a multi-institutional real-world observational study. Open Forum Infect Dis.

[12] Mavrakanas T. A., Fournier M. A., Clairoux S.. (2017). Neutropenia in kidney and liver transplant recipients: risk factors and outcomes. Clin Transplant.

[13] Turzhitsky V., Raval A. D., Moise P., Merchant S. (2022). Neutropenia and associated infectious complications among kidney transplant recipients receiving valganciclovir prophylaxis in the United States: an administrative claims database study. Open Forum Infect Dis.

[14] Hellemans R., Wijtvliet V., Bergs K.. (2021). A split strategy to prevent cytomegalovirus after kidney transplantation using prophylaxis in serological high-risk patients and a pre-emptive strategy in intermediate-risk patients: combining the best of two options?. Transpl Infect Dis.

[15] Hurst F. P., Belur P., Nee R.. (2011). Poor outcomes associated with neutropenia after kidney transplantation: analysis of United States Renal Data System. Transplantation.

[16] Liang X., Famure O., Li Y., Kim S.J. (2018). Incidence and risk factors for leukopenia in kidney transplant recipients receiving valganciclovir for cytomegalovirus prophylaxis. Prog Transplant.

[17] Ingold L., Halter J., Martinez M.. (2021). Short-and long-term impact of neutropenia within the first year after kidney transplantation. Transplant Int.

[18] Brar S., Berry R., Raval A. D., Tang Y., Vincenti F., Skartsis N. (2022). Outcomes among CMV-mismatched and highly sensitized kidney transplants recipients who develop neutropenia. Clin Transplant.

[19] Razonable R. R., Humar A. (2019). Cytomegalovirus in solid organ transplant recipients-guidelines of the American Society of Transplantation Infectious Diseases Community of Practice. Clin Transplant.

[20] Russo A., Pierce D., Christensen R.. (2025). Efficacy and safety comparison of letermovir versus valganciclovir for use in cytomegalovirus prophylaxis in adult kidney transplant recipients. Am J Transplant.

[21] Halim M. A., Al-Otaibi T., Gheith O.. (2016). Efficacy and safety of low-dose versus standard-dose valganciclovir for prevention of cytomegalovirus disease in intermediate-risk kidney transplant recipients. Exp Clin Transplant.

[22] Bureau of Labor Statistics Consumer Price indices for medical care (CPI–M). Current Series US Medical Care, 1982-84=100 - CUUR0000SAM, Medical care in the US city average, all urban consumers, not seasonally adjusted.

[23] Bureau of Labor Statistics Consumer Price Index (CPI) for prescription drugs. Not seasonally adjusted, U.S. city average.

[24] Boulay H., Oger E., Cantarovich D.. (2021). Among CMV-positive renal transplant patients receiving non-T-cell depleting induction, the absence of CMV disease prevention is a safe strategy: a retrospective cohort of 372 patients. Transpl Infect Dis.

[25] Turzhitsky V., Raval A., Moise P., Merchant S. (2022). FR−PO829: Healthcare utilization and costs associated with neutropenia/leukopenia in kidney transplant recipients receiving valganciclovir prophylaxis: an administrative claims database study.

[26] Raval A., Turzhitsky V., Fazio-Eynullayeva E., Jin H., Merchant S. (2022). FR−PO849: Health care resource utilization associated with post-transplant neutropenia and leukopenia among kidney transplant recipients: a real-world evidence study.

[27] Grossi P. A., Kamar N., Saliba F.. (2022). Cytomegalovirus management in solid organ transplant recipients: a pre-COVID-19 survey from the working group of the European Society for Organ Transplantation. Transpl Int.

[28] Gokhale M., Stürmer T., Buse J. B. (2020). Real-world evidence: The devil is in the detail. Diabetologia.

[29] Liu F., Panagiotakos D. (2022). Real-world data: a brief review of the methods, applications, challenges and opportunities. BMC Med Res Methodol.

[30] OPTN National data: Transplants in the U.S. by recipient age.

[31] Li Q., Turzhitsky V., Moise P., Jin H., Brzozowski K., Kolobova I. (2025). Healthcare resource utilization associated with leukopenia and neutropenia in kidney transplant recipients receiving valganciclovir in the United States. J Health Econ Outcomes Res.

