# Supplementary material for: Short and Long-Term Outcomes in Kidney Transplant Recipients with Neutropenia or Leukopenia Following CMV Prophylaxis
Source: J Health Econ Outcomes Res. 2026 May 12;13(1):155–67. doi: 10.36469/001c.159410 (PMC13175152; doi:10.36469/001c.159410)
Supplement: Online Supplementary Material [file jheor_2026_13_1_159410_344334.pdf]

Journal of Health Economics and Outcomes Research

## Online Supplementary Material

Short- and Long-Term Outcomes in Kidney Transplant Recipients with Neutropenia or Leukopenia Following CMV Prophylaxis. *JHEOR*. 2026;13(1):155-167. doi:10.36469/jheor.2026.159410

**Table S1: Candidate Variables Used for LASSO Selection of Factors Associated with Neutropenia or Leukopenia** ........ **1**

**Table S2: Baseline Characteristics and Valganciclovir Use up to 1 Year Post-transplant** ........ **4**

**Table S3: Assessment of Potential Post-transplant Neutropenia or Leukopenia Risk Factors Among Individuals Undergoing First Kidney Transplant** ........ **7**

**Table S4: Unadjusted HCRU and Costs 1 Year Post-transplant Among Those with and without Neutropenia or Leukopenia** ........ **8**

**Table S5: Unadjusted HCRU and Costs 1 Year Post-transplant Among Those with and without Neutropenia** ........ **12**

**Table S6: Unadjusted HCRU and Costs 1 Year Post-transplant Among Those with and without Leukopenia** ........ **15**

**Table S7: Differences in Adjusted Costs Between Those with and without Neutropenia and with and without Leukopenia, 1 Year Post-transplant, from Multivariable Models for Clinical Outcomes of Interest** ........ **18**

**Table S8: Unadjusted Costs by Clinical Outcome Event 2 to 5 Years Post-transplant** ........ **19**

**Figure S1: Unadjusted Median HCRU Costs Associated with Neutropenia or Leukopenia (A), Neutropenia Alone (B), and Leukopenia Alone (C) After 1 Year Follow-up** ........ **20**

**Figure S2: Unadjusted Median HCRU Costs Assoiated with Neutropenia or Leukopenia (A), Neutropenia Alone (B), and Leukopenia Alone (C) After 2- to 5-Year Follow-up** ........ **21**

This supplementary material has been provided by the authors to give readers additional information about their work.

This is an open-access article distributed under the terms of the Creative Commons Attribution 4.0 International License (CCBY-4.0). View this license's legal deed at http://creativecommons.org/licenses/by/4.0 and legal code at http://creativecommons.org/licenses/by/4.0/legalcode for more information.

**Table S1. Candidate Variables Used for LASSO Selection of Factors Associated with Neutropenia or Leukopenia**

| **Risk Factors** |
|---|
| **Demographic Characteristics** |
| **Age categories, years (ref= 18-44)** |
| ≥45 |
| ≥65 |
| ≥75 |
| **Gender** |
| Male (reference) |
| Female |
| **Geographic Region-Organ Procurement and Transplantation Network (OPTN) *** |
| Region 1 |
| Region 2 |
| Region 3 |
| Region 4 |
| Region 5 |
| Region 6 |
| Region 7 |
| Region 8 |
| Region 9 |
| Region 10 |
| Region 11 |
| Unknown |
| **Primary payer type** |
| Commercial health insurance (reference) |
| Private Medicare supplemental coverage |
| **Baseline Clinical Characteristics** |
| **Charlson Comorbidity Index (CCI)**[1] |
| CCI>=1 |
| CCI>=2 |
| CCI>=3 |
| CCI>=4 |
| **Comorbidities (**0=reference**)** |
| Congestive heart failure |
| Dementia |
| Chronic pulmonary disease |
| Rheumatologic disease |
| Mild liver disease |
| Diabetes with chronic complications |
| Diabetes (uncomplicated) |
| Diabetes (any) |
| Hemiplegia or paraplegia |
| Any malignancy, including leukemia and lymphoma |
| Moderate or severe liver disease |
| Metastatic solid tumor |
| AIDS/HIV |
| **Calendar year of transplant (ref= 2012)** |
| ≥2013 |

| |
|---|
| ≥2014 |
| ≥2015 |
| ≥2016 |
| ≥2017 |
| ≥2018 |
| ≥2019 |
| ≥2020 |
| ≥2021 |
| **Time from first ESRD diagnosis to KT (Ref =">=12 months")** |
| >= 3 months |
| >=6 months |
| >=12 months |
| Unavailable |
| **Co-medications** |
| **Steroids** |
| Prednisone |
| Methylprednisolone |
| Prednisone or methylprednisolone |
| **Immunosuppressants** |
| Anti-thymocyte globulin-ATG |
| Alemtuzumab |
| Basiliximab |
| NRATS OKT3 (orthoclone, muromonab) |
| Rituximab |
| Cyclophosphamide |
| Cyclosporin |
| Tacrolimus |
| Sirolimus |
| Everolimus |
| Azathioprine |
| Mycophenolate mofetil |
| Methotrexate |
| BRQ |
| Leflunomide |
| Mizoribine |
| Prednisone |
| Methylprednisolone |
| **HMG-CoA reductase inhibitors** |
| Statins |
| **Healthcare resource utilization (baseline period)** |
| **Emergency department (ED) visits** |
| All-cause ED visits >=4 |
| All-cause ED visits >=2 |
| All-cause ED visits (any) |
| CMV-related ED visits (any) |
| OI-related ED visits (any) |
| Toxicity-related ED visits (any) |
| **Dialysis outpatient visits** |
| Dialysis events (>=160) |
| Dialysis events (>=70) |
| Dialysis events (>=20) |

| |
|---|
| **Other outpatient visits (not ED or dialysis)** |
| All-cause outpatient visits (>=70) |
| All-cause outpatient visits (>=40) |
| All-cause outpatient visits (>=20) |
| OI-related outpatient visits (any) |
| Toxicity-related outpatient visits (any) |
| **Hospitalizations** |
| All-cause hospitalizations visits (>=2) |
| All-cause hospitalizations visits (any) |
| OI-related hospitalizations visits (any) |
| Toxicity-related hospitalizations visits (any) |
| **Prescription counts** |
| All-cause pharmacy prescriptions (>=70) |
| All-cause pharmacy prescriptions (>=40) |
| All-cause pharmacy prescriptions (>=20) |
| CMV-related pharmacy prescriptions (any) |

## Table S2. Baseline[a] Characteristics and Valganciclovir Use up to 1 Year Post-Transplant

| Characteristics | Neutropenia | | | Leukopenia | | |
|---|---|---|---|---|---|---|
| | With Neutropenia | Without Neutropenia | *P* | With Leukopenia | Without Leukopenia | *P* |
| | n=568 | n=2,553 | | n=717 | n=2,404 | |
| **Continuous Age, years** | | | 0.039 | | | 0.0583 |
| Mean (SD) | 51.0 (11.89) | 52.1 (11.91) | | 51.1 (11.99) | 52.1 (11.88) | |
| Min, Max | 18, 85 | 18, 84 | | 18, 85 | 18, 84 | |
| Median (P25, P75) | 52 (43, 60) | 53 (44, 61) | | 52 (44, 60) | 54 (44, 61) | |
| **Age categories, years** | | | 0.3586 | | | 0.5086 |
| 18-44 | 156 (27.5%) | 642 (25.1%) | | 194 (27.1%) | 604 (25.1%) | |
| 45-64 | 357 (62.9%) | 1604 (62.8%) | | 448 (62.5%) | 1513 (62.9%) | |
| 65-74 | 47 (8.3%) | 266 (10.4%) | | 63 (8.8%) | 250 (10.4%) | |
| 75+ | 8 (1.4%) | 41 (1.6%) | | 12 (1.7%) | 37 (1.5%) | |
| **Gender** | | | 0.8607 | | | 0.4431 |
| Male | 340 (59.9%) | 1518 (59.5%) | | 418 (58.3%) | 1440 (59.9%) | |
| Female | 228 (40.1%) | 1035 (40.5%) | | 299 (41.7%) | 964 (40.1%) | |
| **Geographic Region** | | | 0.0371 | | | **<.0001** |
| Northeast | 107 (18.8%) | 467 (18.3%) | | 86 (12.0%) | 488 (20.3%) | |
| North Central | 108 (19.0%) | 554 (21.7%) | | 131 (18.3%) | 531 (22.1%) | |
| South | 287 (50.5%) | 1147 (44.9%) | | 392 (54.7%) | 1042 (43.3%) | |
| West | 66 (11.6%) | 374 (14.6%) | | 105 (14.6%) | 335 (13.9%) | |
| Unknown | 0 | 11 (0.4%) | | 3 (0.4%) | 8 (0.3%) | |
| **Geographic Region-OPTN[b]** | | | 0.0008 | | | **<.0001** |
| Region 1 | 15 (2.6%) | 77 (3.0%) | | 14 (2.0%) | 78 (3.2%) | |
| Region 2 | 40 (7.0%) | 215 (8.4%) | | 49 (6.8%) | 206 (8.6%) | |
| Region 3 | 118 (20.8%) | 423 (16.6%) | | 145 (20.2%) | 396 (16.5%) | |
| Region 4 | 35 (6.2%) | 246 (9.6%) | | 71 (9.9%) | 210 (8.7%) | |
| Region 5 | 41 (7.2%) | 282 (11.0%) | | 73 (10.2%) | 250 (10.4%) | |
| Region 6 | 17 (3.0%) | 72 (2.8%) | | 20 (2.8%) | 69 (2.9%) | |
| Region 7 | 34 (6.0%) | 144 (5.6%) | | 38 (5.3%) | 140 (5.8%) | |
| Region 8 | 15 (2.6%) | 117 (4.6%) | | 30 (4.2%) | 102 (4.2%) | |
| Region 9 | 72 (12.7%) | 247 (9.7%) | | 44 (6.1%) | 275 (11.4%) | |
| Region 10 | 65 (11.4%) | 308 (12.1%) | | 71 (9.9%) | 302 (12.6%) | |
| Region 11 | 108 (19.0%) | 386 (15.1%) | | 145 (20.2%) | 349 (14.5%) | |
| Unknown | 8 (1.4%) | 36 (1.4%) | | 17 (2.4%) | 27 (1.1%) | |
| **Primary payer type** | | | 0.0608 | | | 0.0919 |
| Commercial | 503 (88.6%) | 2184 (85.5%) | | 631 (88.0%) | 2056 (85.5%) | |
| Medicare | 65 (11.4%) | 369 (14.5%) | | 86 (12.0%) | 348 (14.5%) | |
| **Charlson Comorbidity Index[c]** | | | 0.1824 | | | 0.6336 |
| 0 | 187 (32.9%) | 822 (32.2%) | | 234 (32.6%) | 775 (32.2%) | |
| 1–2 | 186 (32.7%) | 755 (29.6%) | | 206 (28.7%) | 735 (30.6%) | |
| 3–4 | 141 (24.8%) | 663 (26.0%) | | 196 (27.3%) | 608 (25.3%) | |
| ≥5 | 54 (9.5%) | 313 (12.3%) | | 81 (11.3%) | 286 (11.9%) | |
| **Comorbidities[d]** | | | | | | |
| Diabetes with complications | 213 (37.5%) | 990 (38.8%) | 0.5714 | 273 (38.1%) | 930 (38.7%) | 0.7683 |
| Congestive heart disease | 87 (15.3%) | 436 (17.1%) | 0.321 | 106 (14.8%) | 417 (17.3%) | 0.1237 |
| Diabetes | 76 (13.4%) | 474 (18.6%) | 0.0033 | 95 (13.2%) | 455 (18.9%) | 0.0005 |
| Mild liver disease | 66 (11.6%) | 300 (11.8%) | 1 | 97 (13.5%) | 269 (11.2%) | 0.0858 |
| Chronic pulmonary disease | 61 (10.7%) | 279 (10.9%) | 0.9407 | 78 (10.9%) | 262 (10.9%) | 1 |
| Cancer | 37 (6.5%) | 193 (7.6%) | 0.4248 | 47 (6.6%) | 183 (7.6%) | 0.3709 |
| Rheumatological disease | 34 (6.0%) | 125 (4.9%) | 0.2916 | 49 (6.8%) | 110 (4.6%) | 0.0198 |

| Characteristics | Neutropenia | | | Leukopenia | | |
|---|---|---|---|---|---|---|
| | With Neutropenia | Without Neutropenia | *P* | With Leukopenia | Without Leukopenia | *P* |
| | n=568 | n=2,553 | | n=717 | n=2,404 | |
| Moderate liver disease | 6 (1.1%) | 44 (1.7%) | 0.3539 | 11 (1.5%) | 39 (1.6%) | 1 |
| **Calendar year of transplant** | | | **<.0001** | | | **<.0001** |
| 2012 | 47 (8.3%) | 369 (14.5%) | | 54 (7.5%) | 362 (15.1%) | |
| 2013 | 29 (5.1%) | 309 (12.1%) | | 62 (8.6%) | 276 (11.5%) | |
| 2014 | 48 (8.5%) | 291 (11.4%) | | 75 (10.5%) | 264 (11.0%) | |
| 2015 | 56 (9.9%) | 315 (12.3%) | | 70 (9.8%) | 301 (12.5%) | |
| 2016 | 88 (15.5%) | 278 (10.9%) | | 86 (12.0%) | 280 (11.6%) | |
| 2017 | 75 (13.2%) | 282 (11.0%) | | 92 (12.8%) | 265 (11.0%) | |
| 2018 | 64 (11.3%) | 190 (7.4%) | | 74 (10.3%) | 180 (7.5%) | |
| 2019 | 85 (15.0%) | 248 (9.7%) | | 98 (13.7%) | 235 (9.8%) | |
| 2020 | 60 (10.6%) | 217 (8.5%) | | 85 (11.9%) | 192 (8.0%) | |
| 2021 | 16 (2.8%) | 54 (2.1%) | | 21 (2.9%) | 49 (2.0%) | |
| **Induction therapy[e]** | | | | | | |
| Anti-thymocyte Globulin-ATG | 38 (6.7%) | 102 (4.0%) | 0.005 | 48 (6.7%) | 92 (3.8%) | 0.0011 |
| Alemtuzumab | 0 | 1 (0.0%) | 1 | 1 (0.1%) | 0 | 0.2297 |
| Basiliximab | 19 (3.3%) | 43 (1.7%) | 0.0103 | 17 (2.4%) | 45 (1.9%) | 0.4006 |
| NRATS OKT3 (Orthoclone, Muromonab)[f] | 0 | 0 | | 0 | 0 | |
| Rituximab | 12 (2.1%) | 22 (0.9%) | 0.0094 | 13 (1.8%) | 21 (0.9%) | 0.0334 |
| Cyclophosphamide | 1 (0.2%) | 7 (0.3%) | 1 | 1 (0.1%) | 7 (0.3%) | 0.6909 |
| **Maintenance therapy[e]** | | | | | | |
| Cyclosporine | 12 (2.1%) | 86 (3.4%) | 0.1206 | 17 (2.4%) | 81 (3.4%) | 0.1785 |
| Tacrolimus | 398 (70.1%) | 1663 (65.1%) | 0.0248 | 491 (68.5%) | 1570 (65.3%) | 0.1155 |
| Sirolimus | 1 (0.2%) | 29 (1.1%) | 0.034 | 7 (1.0%) | 23 (1.0%) | 0.9624 |
| Everolimus | 1 (0.2%) | 8 (0.3%) | 1 | 5 (0.7%) | 4 (0.2%) | 0.0346 |
| Azathioprine | 15 (2.6%) | 36 (1.4%) | 0.0364 | 16 (2.2%) | 35 (1.5%) | 0.1505 |
| Mycophenolate mofetil | 311 (54.8%) | 1,434 (56.2%) | 0.5388 | 409 (57.0%) | 1,336 (55.6%) | 0.4868 |
| Methotrexate | 0 | 4 (0.2%) | 1 | 0 | 4 (0.2%) | 0.5798 |
| BRQ[g] | 0 | 0 | | 0 | 0 | |
| Leflunomide | 0 | 3 (0.1%) | 1 | 1 (0.1%) | 2 (0.1%) | 0.5431 |
| Mizoribine[g] | 0 | 0 | | 0 | 0 | |
| Prednisone | 379 (66.7%) | 1,794 (70.3%) | 0.0966 | 496 (69.2%) | 1,677 (69.8%) | 0.7663 |
| Methylprednisolone | 80 (14.1%) | 278 (10.9%) | 0.0307 | 109 (15.2%) | 249 (10.4%) | 0.0004 |
| **HMG-CoA reductase inhibitors** | | | | | | |
| Statins | 269 (47.4%) | 1252 (49.0%) | 0.4685 | 341 (47.6%) | 1180 (49.1%) | 0.4732 |
| **VGCV use post-transplant[h]** | | | | | | |
| **Duration of VGCV use** | | | 0.1399 | | | 0.2519 |
| <100 days | 308 (54.2%) | 1,465 (57.4%) | | 420 (58.6%) | 1,353 (56.3%) | |
| Between 100 days and 199 days | 208 (36.6%) | 826 (32.4%) | | 236 (32.9%) | 798 (33.2%) | |
| 200+ days | 52 (9.2%) | 262 (10.3%) | | 61 (8.5%) | 253 (10.5%) | |
| **VGCV Initiation & discontinuation** | | | | | | |
| **Days from index to first VGCV Fill-450 mg/day dosage** | | | 0.4689 | | | 0.0005 |
| Mean (SD) | 5.5 (7.66) | 4.9 (6.52) | | 5.9 (7.56) | 4.7 (6.47) | |

| Characteristics | Neutropenia | | | Leukopenia | | |
|---|---|---|---|---|---|---|
| | With Neutropenia | Without Neutropenia | *P* | With Leukopenia | Without Leukopenia | *P* |
| | n=568 | n=2,553 | | n=717 | n=2,404 | |
| Min, Max | 0, 30 | 0, 30 | | 0, 30 | 0, 30 | |
| Median (P25, P75) | 3 (1, 5) | 3 (1, 5) | | 3 (2, 5) | 3 (1, 4) | |
| **Days from index to first VGCV Fill-900mg/day dosage** | | | 0.2243 | | | 0.9591 |
| Mean (SD) | 4.7 (6.70) | 4.6 (5.91) | | 4.6 (6.11) | 4.6 (6.08) | |
| Min, Max | 0, 30 | 0, 30 | | 0, 30 | 0, 30 | |
| Median (P25, P75) | 2 (1, 4) | 3 (1, 5) | | 2 (1, 4) | 3 (1, 4) | |
| **Discontinued VGCV with a gap ≥15 days** | | | **<.0001** | | | **<.0001** |
| Yes | 268 (47.2%) | 846 (33.1%) | | 335 (46.7%) | 779 (32.4%) | |

Abbreviations: AIDS, acquired immunodeficiency syndrome; ATG, anti-thymocyte globulin; CDHP, consumer directed health plan; EPO, exclusive provider organization; HCPCS, healthcare common procedure coding system; HDHP, high deductible health plan; HMO, health maintenance organization; NDC, national drug code; OPTN, Organ Procurement and Transplantation Network; POS, point of service; PPO, preferred provider organization; SD, standard deviation; VGCV, valganciclovir.

[a] One-year prior to kidney transplant.

[b] Unknown region – No state-level data available, or Vermont.

[c] Excludes renal disease.

[d] Comorbidities with a prevalence <1% not reported.

[e] Include baseline period, index hospitalization and up to 14 days post kidney transplant time period.

[f] NDC and HCPCS codes were not present in baseline period, index hospitalization and up to 14 days post kidney transplant time period.

[g] NDC and HCPCS codes were unavailable.

[h] Data relate to 1 year post-kidney transplant.

**Table S3. Assessment of Potential Post-Transplant Neutropenia or Leukopenia Risk Factors Among Individuals Undergoing First Kidney Transplant**

| **Risk factors** | **OR** | **95% CI** | **P-value[b]** |
|---|---|---|---|
| **HCRU** *(relative to no named factor)* | | | |
| Toxicity-related claim[a] | 1.696 | 1.174, 2.451 | 0.0049 |
| **OPTN region** (*relative to OPTN region 3)* | | | |
| Region 1 | 0.708 | 0.426, 1.177 | 0.1832 |
| Region 10 | 0.788 | 0.587, 1.058 | 0.1133 |
| Region 11 | 1.002 | 0.771, 1.301 | 0.9896 |
| Region 2 | 0.585 | 0.415, 0.824 | 0.0022 |
| Region 4 | 0.834 | 0.61, 1.142 | 0.2583 |
| Region 5 | 0.685 | 0.5, 0.938 | 0.0183 |
| Region 6 | 0.837 | 0.503, 1.39 | 0.4912 |
| Region 7 | 0.63 | 0.428, 0.928 | 0.0193 |
| Region 8 | 0.553 | 0.355, 0.863 | 0.0091 |
| Region 9 | 0.593 | 0.433, 0.813 | 0.0012 |
| Unknown | 1.287 | 0.676, 2.45 | 0.4429 |
| **Comorbidities** *(relative to no named factor)* | | | |
| Rheumatologic disease | 1.481 | 1.049, 2.091 | 0.0256 |
| **Year of transplant** *(relative to 2012)* | | | |
| 2013 | 1.155 | 0.802, 1.663 | 0.4385 |
| 2014 | 1.734 | 1.224, 2.455 | 0.0019 |
| 2015 | 1.604 | 1.138, 2.261 | 0.007 |
| 2016 | 2.549 | 1.825, 3.559 | **<.0001** |
| 2017 | 2.264 | 1.612, 3.179 | **<.0001** |
| 2018 | 2.644 | 1.841, 3.797 | **<.0001** |
| 2019 | 2.901 | 2.07, 4.067 | **<.0001** |
| 2020 | 2.552 | 1.789, 3.642 | **<.0001** |
| 2021 | 3.049 | 1.765, 5.266 | **<.0001** |
| **Maintenance therapy** *(relative to no named factor)* | | | |
| Prednisone or methylprednisolone | 0.736 | 0.614, 0.883 | 0.0009 |
| Cyclosporine | 0.617 | 0.37, 1.028 | 0.0637 |
| Tacrolimus | 1.212 | 1.019, 1.443 | 0.0301 |
| **Induction therapy** *(relative to no named factor)* | | | |
| Rituximab | 2.144 | 1.051, 4.373 | 0.0359 |

Abbreviations: CI, confidence interval; ED, emergency department; HCRU, healthcare resource utilization; OPTN, Organ Procurement and Transplantation Network; OR, odds ratio.

[a] Toxicity = any toxicity-related (neutropenia, leukopenia or thrombocytopenia) claim at baseline (either ED, outpatient, or inpatient).

[b] Chi-squared P-value reported.

## Table S4. Unadjusted HCRU and Costs 1 Year Post-Transplant Among Those with and without Neutropenia or Leukopenia

| HCRU at follow-up | With neutropenia or leukopenia n=1,013 | | | | Without neutropenia or leukopenia n=2,108 | | | | P-value[a] (Patients with event) | P-value[b] (Utilization per patient) | P-value[c] (Costs per patient) |
|---|---|---|---|---|---|---|---|---|---|---|---|
| | Patients with event (% of entire cohort) | Mean utilization per patient with resource use (SD), 95% CI | Mean cost per patient with resource use (SD) ($) | Median cost per patient with resource use (IQR) ($) | Patients with event (% of entire cohort) | Mean utilization per patient with resource use (SD), 95% CI | Mean cost per patient with resource use (SD) ($) | Median cost per patient with resource use (IQR) ($) | | | |
| **ED visits** | | | | | | | | | | | |
| All-cause ED visits | 615 (60.7%) | 2.73 (2.66), (2.56–2.89) | 5,649 (11,455) | 2,477 (891–5,897) | 1,120 (53.1%) | 2.52 (2.56), (2.41–2.63) | 5,279 (19,797) | 2,071 (844–5,056) | **<.0001** | 0.0065 | 0.0542 |
| CMV-related ED visits | 56 (5.53%) | 1.21 (0.494), (1.18–1.24) | 3,199 (6,083) | 1,028 (360–2,365) | 19 (0.901%) | 1.32 (0.582), (1.29–1.34) | 2,281 (3,617) | 1,529 (468–2,513) | **<.0001** | 0.4358 | 0.7102 |
| CMV infection | 27 (2.67%) | 1.48 (0.893), (1.43–1.54) | 4,018 (6,110) | 2,161 (939–4,932) | 19 (0.901%) | 1.58 (1.12), (1.53–1.63) | 2,688 (3,777) | 1,644 (322–3,035) | 0.0001 | 0.8797 | 0.3374 |
| csCMVi (ICD-10 codes + treatment change) | 23 (2.27%) | 1.09 (0.288), (1.07–1.1) | 2,908 (5,763) | 906 (306–2,262) | 10 (0.474%) | 1.1 (0.316), (1.09–1.11) | 1,559 (1,715) | 1,099 (349–2,089) | **<.0001** | 0.9373 | 0.8601 |
| CMV disease (ICD-10 codes + treatment change) | 13 (1.28%) | 1.15 (0.376), (1.13–1.18) | 2,973 (4,583) | 788 (358–2,887) | 5 (0.237%) | 1.2 (0.447), (1.18–1.22) | 1,168 (869) | 1,449 (468–1,777) | 0.0003 | 0.8788 | 0.6221 |
| Opportunistic infection-related ED visits | 135 (13.3%) | 1.44 (1.1), (1.37–1.51) | 4,073 (9,171) | 1,303 (310–3,440) | 205 (9.72%) | 1.4 (0.746), (1.37–1.44) | 3,665 (18,254) | 1,392 (437–2,926) | 0.0025 | 0.4155 | 0.8741 |
| Toxicity-related ED visits | 153 (15.1%) | 1.35 (0.823), (1.3–1.4) | 3,454 (7,830) | 1,496 (602–3,289) | 20 (0.949%) | 1.2 (0.696), (1.17–1.23) | 3,639 (7,875) | 877 (169–3,236) | **<.0001** | 0.292 | 0.2075 |
| **Dialysis outpatient visits** | | | | | | | | | | | |
| All-cause dialysis visits | 252 (24.9%) | 6.3 (22.4), (4.92–7.68) | 12,734 (105,797) | 536 (244–2,201) | 533 (25.3%) | 3.45 (11.8), (2.95–3.95) | 4,437 (24,570) | 427 (263–1,251) | 0.8057 | 0.0032 | 0.0879 |
| **Other outpatient visits (not ED or dialysis)** | | | | | | | | | | | |
| All-cause other outpatient visits | 1,013 (100%) | 55.5 (22.6), (54.1–56.9) | 52,926 (95,599) | 30,540 (16,434–60,164) | 2,108 (100%) | 49.3 (22.8), (48.4–50.3) | 45,409 (96,961) | 27,514 (14,598–50,967) | - | **<.0001** | 0.0003 |
| CMV-related other outpatient visits | 301 (29.7%) | 7.17 (9.74), (6.57–7.77) | 8,467 (19,906) | 1,838 (567–7,373) | 232 (11%) | 4.85 (7.28), (4.54–5.16) | 3,572 (8,676) | 1,187 (437–3,237) | **<.0001** | 0.0003 | 0.0008 |

| HCRU at follow-up | With neutropenia or leukopenia n=1,013 | | | | Without neutropenia or leukopenia n=2,108 | | | | P-value[a] (Patients with event) | P-value[b] (Utilization per patient) | P-value[c] (Costs per patient) |
|---|---|---|---|---|---|---|---|---|---|---|---|
| | Patients with event (% of entire cohort) | Mean utilization per patient with resource use (SD), 95% CI | Mean cost per patient with resource use (SD) ($) | Median cost per patient with resource use (IQR) ($) | Patients with event (% of entire cohort) | Mean utilization per patient with resource use (SD), 95% CI | Mean cost per patient with resource use (SD) ($) | Median cost per patient with resource use (IQR) ($) | | | |
| CMV infection | 149 (14.7%) | 9.15 (11), (8.47–9.82) | 11,743 (26,271) | 2,455 (619–10,520) | 112 (5.31%) | 8.96 (13.5), (8.39–9.54) | 4,595 (8,244) | 1,806 (423–5,966) | **<.0001** | 0.355 | 0.0461 |
| csCMVi (ICD-10 codes + treatment change) | 80 (7.9%) | 5.98 (6.95), (5.55–6.4) | 5,580 (8,253) | 1,927 (504–6,813) | 44 (2.09%) | 3.16 (5.6), (2.92–3.4) | 2,565 (5,012) | 582 (288–2,083) | **<.0001** | 0.0043 | 0.0092 |
| CMV disease (ICD-10 codes + treatment change) | 46 (4.54%) | 8.33 (9.75), (7.73–8.93) | 10,078 (16,816) | 2,834 (239–11,031) | 14 (0.664%) | 4.29 (5.06), (4.07–4.5) | 2,545 (4,473) | 560 (227–2,337) | **<.0001** | 0.1769 | 0.1306 |
| Opportunistic infection-related other outpatient visits | 429 (42.3%) | 6.22 (9.69), (5.63–6.82) | 7,385 (30,248) | 1,422 (366–5,078) | 603 (28.6%) | 5.32 (9.51), (4.92–5.73) | 7,635 (36,299) | 1,137 (370–4,455) | **<.0001** | 0.1602 | 0.4721 |
| Toxicity-related other outpatient visits | 909 (89.7%) | 3.67 (4.09), (3.42–3.92) | 4,129 (8,653) | 1,448 (519–4,181) | 36 (1.71%) | 2.92 (4.26), (2.73–3.1) | 4,533 (11,231) | 1,187 (342–4,046) | **<.0001** | 0.0164 | 0.3846 |
| **Inpatient hospitalizations** | | | | | | | | | | | |
| All-cause hospitalization visits | 513 (50.6%) | 2.01 (1.49), (1.92–2.1) | 67,072 (97,691) | 37,180 (15,637–78,804) | 796 (37.8%) | 1.64 (1.26), (1.58–1.69) | 66,608 (172,988) | 24,983 (12,611–59,769) | **<.0001** | **<.0001** | **<.0001** |
| CMV-related hospitalization visits | 113 (11.2%) | 1.5 (0.965), (1.44–1.55) | 45,860 (57,690) | 24,596 (12,150–52,723) | 50 (2.37%) | 1.2 (0.452), (1.18–1.22) | 58,596 (123,750) | 27,154 (14,406–56,083) | **<.0001** | 0.0632 | 0.5956 |
| CMV infection | 33 (3.26%) | 1.55 (0.869), (1.49–1.6) | 43,511 (39,596) | 33,517 (12,150–60,962) | 25 (1.19%) | 1.36 (0.638), | 43,962 (44,680) | 31,787 (16,613–58,073) | **<.0001** | 0.4708 | 0.718 |
| csCMVi (ICD-10 codes + treatment change) | 37 (3.65%) | 1.24 (0.435), (1.22–1.27) | 29,465 (34,999) | 17671 (9258 - 32387) | 15 (0.712%) | 1.13 (0.352), | 81,131 (212,627) | 23,095 (10,671–27,885) | **<.0001** | 0.3917 | NA |
| CMV disease (ICD-10 codes + treatment change) | 24 (2.37%) | 1.63 (1.17), (1.55–1.7) | 58,079 (68,646) | 26,129 (10,884 – 83,199) | 8 (0.38%) | 1.13 (0.354), | 61,976 (95,184) | 25,986 (23,239–48,714) | **<.0001** | 0.2365 | 0.8108 |

| HCRU at follow-up | With neutropenia or leukopenia n=1,013 | | | | Without neutropenia or leukopenia n=2,108 | | | | P-value[a] (Patients with event) | P-value[b] (Utilization per patient) | P-value[c] (Costs per patient) |
|---|---|---|---|---|---|---|---|---|---|---|---|
| | Patients with event (% of entire cohort) | Mean utilization per patient with resource use (SD), 95% CI | Mean cost per patient with resource use (SD) ($) | Median cost per patient with resource use (IQR) ($) | Patients with event (% of entire cohort) | Mean utilization per patient with resource use (SD), 95% CI | Mean cost per patient with resource use (SD) ($) | Median cost per patient with resource use (IQR) ($) | | | |
| Opportunistic infection -related hospitalization visits | 195 (19.2%) | 1.47 (0.949), (1.41–1.53) | 59,905 (102,979) | 29,720 (14,696–64,712) | 239 (11.3%) | 1.37 (0.788), (1.33–1.4) | 75,903 (202,448) | 24,759 (12,792–58,140) | **<.0001** | 0.1135 | 0.114 |
| Toxicity-related hospitalization visits | 223 (22%) | 1.27 (0.6), (1.23–1.31) | 44,354 (88,225) | 22,334 (13,474–43,736) | 27 (1.28%) | 1.11 (0.32), (1.1–1.12) | 28,207 (26,629) | 19,684 (9,494–37,626) | **<.0001** | 0.1676 | 0.1882 |
| **All-cause length of stay (days)** | | | | | | | | | | | |
| Total number of hospitalization days | - | 12.8 (15.3), (11.8–13.7) | - | - | - | 10.4 (19.4), (9.59–11.2) | - | - | - | **<.0001** | - |
| Length of stay by visit (days) | - | 6.01 (4.71), (5.6–6.42) | - | - | - | 5.6 (4.63), (5.28–5.92) | - | - | - | 0.004 | - |
| **Medication counts (inpatient or outpatient)** | | | | | | | | | | | |
| CMV-related medical counts | 65 (6.42%) | 4.98 (4.99), (4.68–5.29) | 6,999 (12,059) | 2,292 (853–6,098) | 29 (1.38%) | 6.86 (7.37), (6.55–7.18) | 6,897 (8,287) | 3,286 (1,628–8,487) | **<.0001** | 0.5091 | 0.4687 |
| Toxicity-related medical counts | 447 (44.1%) | 4.73 (7.28), (4.29–5.18) | 37,640 (98,893) | 1,950 (429–25,031) | 497 (23.6%) | 5.48 (11.6), (4.99–5.98) | 57,407 (125,003) | 4,226 (24–51,200) | **<.0001** | 0.0502 | 0.5393 |
| G-CSF-related medical counts | 260 (25.7%) | 3.25 (3.53), (3.03–3.46) | 11,192 (42,363) | 1,264 (480–3,953) | 99 (4.7%) | 3.94 (4.64), (3.74–4.14) | 33,034 (73,564) | 8,426 (1,076–34,672) | **<.0001** | 0.054 | **<.0001** |
| Blood transfusion-related medical counts | 209 (20.6%) | 3.31 (6.79), (2.89–3.72) | 51,932 (100,545) | 9,020 (0–50,508) | 382 (18.1%) | 3.69 (7.06), (3.38–3.99) | 58,298 (108,454) | 1,179 (0–62,814) | 0.0937 | 0.6825 | 0.66 |
| **Prescription pharmacy counts (NDC)** | | | | | | | | | | | |
| All-cause pharmacy prescriptions | 1,013 (100%) | 85.4 (34), (83.3–87.5) | 32,984 (41,431) | 25,013 (14,822–41,183) | 2,108 (100%) | 84.5 (34), (83–85.9) | 30,878 (38,795) | 24,009 (14,999–36,909) | - | 0.4902 | 0.0538 |
| CMV-related pharmacy prescriptions | 1,013 (100%) | 4.85 (2.63), (4.69–5.01) | 11,442 (11,696) | 7,620 (3,723–14,608) | 2,108 (100%) | 4.43 (2.43), (4.32–4.53) | 10,246 (9,245) | 7,487 (4,222–13,584) | - | **<.0001** | 0.5884 |
| Toxicity (G-CSF)-related pharmacy prescriptions | 109 (10.8%) | 2.08 (1.94), (1.96–2.2) | 4,041 (5,314) | 2,422 (1,218–4,628) | 58 (2.75%) | 1.72 (2.81), (1.6–1.84) | 3,946 (6,506) | 1,802 (1,146–4,068) | **<.0001** | 0.0129 | 0.2681 |

| HCRU at follow-up | With neutropenia or leukopenia n=1,013 | | | | Without neutropenia or leukopenia n=2,108 | | | | P-value[a] (Patients with event) | P-value[b] (Utilization per patient) | P-value[c] (Costs per patient) |
|---|---|---|---|---|---|---|---|---|---|---|---|
| | Patients with event (% of entire cohort) | Mean utilization per patient with resource use (SD), 95% CI | Mean cost per patient with resource use (SD) ($) | Median cost per patient with resource use (IQR) ($) | Patients with event (% of entire cohort) | Mean utilization per patient with resource use (SD), 95% CI | Mean cost per patient with resource use (SD) ($) | Median cost per patient with resource use (IQR) ($) | | | |
| **Total** | | | | | | | | | | | |
| Total costs | - | - | 126,474 (176,681) | 82,536 (47,984–143,834) | - | - | 105,363 (165,720) | 68,652 (43,191–111,155) | - | - | **<.0001** |

CI, confidence interval; CMV, cytomegalovirus; csCMVi, clinically significant cytomegalovirus infection; ED, emergency department; G-CSF, granulocyte-colony stimulating factor; HCRU, healthcare resource utilization; ICD, international classification of disease; IQR, interquartile range; NDC, national drug code; SD, standard deviation.
[a] Chi-squared P-value reported for comparison of patients with event (% of entire cohort).
[b] Wilcoxon rank-sum P-value reported for comparison of mean utilization per patient (among those with resource use).
[c] Wilcoxon rank-sum P-value reported for comparison of cost per patient (among those with resource use)

## Table S5. Unadjusted HCRU and Costs 1 Year Post-Transplant among Those with and without Neutropenia

| HCRU at follow-up | With neutropenia n = 568 | | | | Without neutropenia n = 2,553 | | | | P-value[a] (Patients with event) | P-value[b] (Utilization per patient) | P-value[c] (Costs per patient) |
|---|---|---|---|---|---|---|---|---|---|---|---|
| | **Patients with event (% of entire cohort)** | **Mean utilization per patient with resource use (SD), 95% CI** | **Mean cost per patient with resource use (SD) (US$ 2022)** | **Median cost per patient with resource use (IQR) (US$ 2022)** | **Patients with event (% of entire cohort)** | **Mean utilization per patient with resource use (SD), 95% CI** | **Mean cost per patient with resource use (SD) (US$ 2022)** | **Median cost per patient with resource use (IQR) (US$ 2022)** | | | |
| **ED visits** | | | | | | | | | | | |
| All-cause ED visits | 352 (62%) | 2.8 (2.81), (2.56–3.03) | 5,605 (12,555) | 2,358 (936–5,539) | 1,383 (54.2%) | 2.54 (2.54), (2.44–2.64) | 5,361 (18,319) | 2,176 (842–5,215) | 0.0007 | 0.0117 | 0.4401 |
| CMV-related ED visits | 39 (6.87%) | 1.23 (0.485), (1.19–1.27) | 3,725 (6,929) | 1,033 (361–2,575) | 36 (1.41%) | 1.25 (0.554), (1.23–1.27) | 2,145 (3,427) | 1,261 (391–2,070) | **<.0001** | 0.9696 | 0.7827 |
| CMV infection | 18 (3.17%) | 1.44 (0.616), (1.39–1.5) | 3,497 (3,215) | 2,365 (1,271–4,932) | 28 (1.1%) | 1.57 (1.17), (1.53–1.62) | 3,450 (6,299) | 1,583 (385–3,670) | 0.0002 | 0.5884 | 0.1466 |
| csCMVi (ICD-10 codes + treatment change) | 19 (3.35%) | 1.11 (0.315), (1.08–1.13) | 3,201 (6,317) | 648 (306–3,953) | 14 (0.548%) | 1.07 (0.267), (1.06–1.08) | 1,547 (1,506) | 1,353 (349–2,089) | **<.0001** | 0.7699 | 0.6488 |
| CMV disease (ICD-10 codes + treatment change) | 10 (1.76%) | 1.2 (0.422), (1.17–1.23) | 3,493 (5,151) | 1,337 (202–4,722) | 8 (0.313%) | 1.13 (0.354), (1.11–1.14) | 1,195 (811) | 1,119 (568–1,933) | 0.0004 | 0.7312 | 0.8242 |
| Opportunistic infection-related ED visits | 85 (15%) | 1.52 (1.32), (1.41–1.63) | 4,491 (10,913) | 1,263 (412–3,440) | 255 (9.99%) | 1.38 (0.711), (1.36–1.41) | 3,606 (16,499) | 1,392 (360–3,055) | 0.0006 | 0.6923 | 0.8244 |
| Toxicity-related ED visits | 123 (21.7%) | 1.41 (0.896), (1.34–1.49) | 3,620 (8,609) | 1,350 (508–3,380) | 50 (1.96%) | 1.14 (0.495), (1.12–1.16) | 3,120 (5,432) | 1,774 (394–2,884) | **<.0001** | 0.0434 | 0.8316 |
| **Dialysis outpatient visits** | | | | | | | | | | | |
| All-cause dialysis visits | 139 (24.5%) | 7.76 (28.1), (5.45–10.1) | 18,421 (141,443) | 492 (236–2,014) | 646 (25.3%) | 3.63 (11.8), (3.18–4.09) | 4,665 (23,621) | 449 (263–1,433) | 0.6795 | 0.1015 | 0.6049 |
| **Other outpatient visits (not ED or dialysis)** | | | | | | | | | | | |
| All-cause other outpatient visits | 568 (100%) | 57.3 (23), (55.5–59.2) | 55,463 (115,332) | 31,850 (16,618–61,794) | 2,553 (100%) | 50 (22.7), (49.1–50.9) | 46,155 (91,816) | 27,920 (14,868–52,017) | - | **<.0001** | 0.0016 |
| CMV-related other outpatient visits | 198 (34.9%) | 8.19 (10.5), (7.33–9.05) | 8805 (17595) | 2,530 (719–9,854) | 335 (13.1%) | 4.97 (7.44), (4.68–5.26) | 4,878 (15,134) | 1,180 (439–3,726) | **<.0001** | **<.0001** | **<.0001** |
| CMV infection | 90 (15.8%) | 10.6 (12.1), (9.58–11.6) | 13,856 (27,601) | 2,957 (732–15,735) | 171 (6.7%) | 8.27 (12.1), (7.81–8.74) | 5,949 (15,618) | 1,770 (415–6,382) | **<.0001** | 0.087 | 0.0114 |
| csCMVi (ICD-10 codes + treatment change) | 62 (10.9%) | 6.79 (7.41), (6.18–7.4) | 6,227 (7,814) | 2,762 (730–9,500) | 62 (2.43%) | 3.16 (5.19), (2.96–3.36) | 2,793 (6,564) | 543 (262–1,720) | **<.0001** | 0.0002 | **<.0001** |

| HCRU at follow-up | With neutropenia n = 568 | | | | Without neutropenia n = 2,553 | | | | P-value[a] (Patients with event) | P-value[b] (Utilization per patient) | P-value[c] (Costs per patient) |
|---|---|---|---|---|---|---|---|---|---|---|---|
| | **Patients with event (% of entire cohort)** | **Mean utilization per patient with resource use (SD), 95% CI** | **Mean cost per patient with resource use (SD) (US$ 2022)** | **Median cost per patient with resource use (IQR) (US$ 2022)** | **Patients with event (% of entire cohort)** | **Mean utilization per patient with resource use (SD), 95% CI** | **Mean cost per patient with resource use (SD) (US$ 2022)** | **Median cost per patient with resource use (IQR) (US$ 2022)** | | | |
| CMV disease (ICD-10 codes + treatment change) | 35 (6.16%) | 9.17 (10.4), (8.32–10) | 10,265 (17,038) | 3,079 (360–11,249) | 25 (0.979%) | 4.88 (5.93), (4.65–5.11) | 5,597 (11,914) | 642 (211–3,873) | **<.0001** | 0.1645 | 0.112 |
| Opportunistic infection-related other outpatient visits | 258 (45.4%) | 6.71 (10.7), (5.83–7.58) | 5,777 (13,820) | 1,499 (397–4,769) | 774 (30.3%) | 5.36 (9.18), (5–5.72) | 8,116 (38,316) | 1,141 (361–4,507) | **<.0001** | 0.0526 | 0.3278 |
| Toxicity-related other outpatient visits | 514 (90.5%) | 4.6 (4.88), (4.2–5) | 5,266 (10,560) | 1,832 (667–5,148) | 431 (16.9%) | 2.5 (2.45), (2.41–2.6) | 2,807 (5,661) | 1,039 (363–2,864) | **<.0001** | **<.0001** | **<.0001** |
| **Inpatient hospitalizations** | | | | | | | | | | | |
| All-cause hospitalization visits | 312 (54.9%) | 2.03 (1.55), (1.9–2.15) | 68,055 (95,083) | 34,867 (16,764–81,458) | 997 (39.1%) | 1.71 (1.3), (1.66–1.76) | 66,394 (161,146) | 26,755 (13,037–64,471) | **<.0001** | **<.0001** | 0.0017 |
| CMV related hospitalization visits | 75 (13.2%) | 1.52 (1.04), (1.43–1.61) | 51,076 (58,377) | 29,114 (13,752–81,059) | 88 (3.45%) | 1.31 (0.632), (1.28–1.33) | 48,651 (100,364) | 24,232 (10,772–49,756) | **<.0001** | 0.2577 | 0.1993 |
| CMV infection | 21 (3.7%) | 1.38 (0.74), (1.32–1.44) | 51,032 (43,734) | 37,938 (21,122–65,196) | 37 (1.45%) | 1.51 (0.804), (1.48–1.54) | 39,547 (40,161) | 26,181 (11,060–52,610) | 0.0003 | 0.5379 | 0.1793 |
| csCMVi (ICD-10 codes + treatment change) | 25 (4.4%) | 1.28 (0.458), (1.24–1.32) | - | - | 27 (1.06%) | 1.15 (0.362), (1.13–1.16) | - | - | **<.0001** | 0.2547 | - |
| CMV disease (ICD-10 codes + treatment change) | 15 (2.64%) | 1.87 (1.41), (1.75–1.98) | 74,025 (79,212) | 34,157 (11,304–115,497) | 17 (0.666%) | 1.18 (0.393), (1.16–1.19) | 45,843 (69,697) | 23,867 (11,859–31,948) | **<.0001** | 0.1043 | 0.3648 |
| Opportunistic infection - related hospitalization visits | 122 (21.5%) | 1.58 (1.13), (1.49–1.67) | 61,308 (100,912) | 29,646 (14,616–65,414) | 312 (12.2%) | 1.35 (0.728), (1.32–1.38) | 71,611 (184,598) | 27,132 (13,031–59,120) | **<.0001** | 0.037 | 0.2867 |
| Toxicity-related hospitalization visits | 161 (28.3%) | 1.3 (0.66), (1.24–1.35) | 43,203 (76,725) | 25,260 (14,101–45,460) | 89 (3.49%) | 1.17 (0.376), (1.15–1.18) | 41,538 (95,978) | 18,095 (10,941–40,562) | **<.0001** | 0.1803 | 0.1133 |
| **All-cause length of stay (days)** | | | | | | | | | | | |
| Total number of hospitalization days | - | 13 (16.2), (11.7–14.3) | - | - | - | 10.8 (18.4), (10.1–11.5) | - | - | - | **<.0001** | - |
| Length of stay by visit (days) | - | 6.1 (5.24), (5.52–6.69) | - | - | - | 5.65 (4.46), (5.37–5.93) | - | - | - | 0.0582 | - |

| HCRU at follow-up | With neutropenia n = 568 | | | | Without neutropenia n = 2,553 | | | | P-value[a] (Patients with event) | P-value[b] (Utilization per patient) | P-value[c] (Costs per patient) |
|---|---|---|---|---|---|---|---|---|---|---|---|
| | **Patients with event (% of entire cohort)** | **Mean utilization per patient with resource use (SD), 95% CI** | **Mean cost per patient with resource use (SD) (US$ 2022)** | **Median cost per patient with resource use (IQR) (US$ 2022)** | **Patients with event (% of entire cohort)** | **Mean utilization per patient with resource use (SD), 95% CI** | **Mean cost per patient with resource use (SD) (US$ 2022)** | **Median cost per patient with resource use (IQR) (US$ 2022)** | | | |
| **Medication counts (inpatient or outpatient)** | | | | | | | | | | | |
| CMV-related medical counts | 46 (8.1%) | 5.2 (5.45), (4.75–5.64) | 6,291 (10,034) | 2,347 (918–5,228) | 48 (1.88%) | 5.92 (6.25), (5.67–6.16) | 7,615 (11,902) | 3,212 (731–9,496) | **<.0001** | 0.9029 | 0.7336 |
| Toxicity-related medical counts | 293 (51.6%) | 4.79 (7.29)[b], (4.19–5.39) | 29,669 (85,604) | 1,794 (449–14,338) | 651 (25.5%) | 5.28 (10.7), (4.86–5.7) | 56,346 (123,573) | 3,699 (36–50,166) | **<.0001** | 0.0173 | 0.1493 |
| G-CSF-related medical counts | 205 (36.1%) | 3.19 (3.39), (2.91–3.46) | 5,553 (22,398) | 1,151 (429–3,312) | 154 (6.03%) | 3.77 (4.42), (3.6–3.94) | 32,740 (75,061) | 6,475 (1,033–26,004) | **<.0001** | 0.1616 | **<.0001** |
| Blood transfusion-related medical counts | 119 (21%) | 3.26 (6.67), (2.71–3.81) | 55926(110837) | 9,419 (11–60,915) | 472 (18.5%) | 3.63 (7.04), (3.35–3.9) | 56,086 (10,4473) | 1,376 (0–58,697) | 0.1755 | 0.361 | 0.6682 |
| **Prescription pharmacy counts (NDC)** | | | | | | | | | | | |
| All-cause pharmacy prescriptions | 568 (100%) | 86.1 (32.2), (83.5–88.8) | 34,008 (49,737) | 25,239 (15,231–42,079) | 2553 (100%) | 84.5 (34.4), (83.1–85.8) | 31,018 (37,057) | 24,185 (14,879–37,446) | - | 0.1798 | 0.0901 |
| CMV-related pharmacy prescriptions | 568 (100%) | 5.08 (2.67), (4.86–5.3) | 11,791 (11,759) | 7902 (3638–15340) | 2553 (100%) | 4.45 (2.45), (4.36–4.55) | 10,377 (9,701) | 7,455 (4,167–13,596) | - | **<.0001** | 0.2816 |
| Toxicity (G-CSF)-related pharmacy prescriptions | 93 (16.4%) | 2.11 (1.56), (1.98–2.24) | 4,063 (4,880) | 2,527 (1,460–4,658) | 74 (2.9%) | 1.77 (2.94), (1.66–1.88) | 3,938 (6,694) | 1,619 (924–3,871) | **<.0001** | 0.0003 | 0.0308 |
| **Total** | | | | | | | | | | | |
| Total costs | - | - | 134,835 (210,742) | 85,348 (50,388–144,383) | - | - | 107,183 (158,638) | 70,148 (43,453–116,633) | - | - | **<.0001** |

Abbreviations: CI, confidence interval; CMV, cytomegalovirus; csCMVi, clinically significant cytomegalovirus infection; ED, emergency department; G-CSF, granulocyte-colony stimulating factor; HCRU, healthcare resource utilization; ICD, international classification of disease; IQR, interquartile range; NDC, national drug code; SD, standard deviation.
[a] Chi-squared P-value reported for comparison of patients with event (% of entire cohort).
[b] Wilcoxon rank-sum P-value reported for comparison of mean utilization per patient (among those with resource use).
[c] Wilcoxon rank-sum P-value reported for comparison of cost per patient (among those with resource use).
CMV = cytomegalovirus; csCMVi = clinically significant cytomegalovirus infection; G-CSF = granulocyte-colony stimulating factor.

## Table S6. Unadjusted HCRU and Costs 1 Year Post-Transplant Among Those with and without Leukopenia

| HCRU at follow-up | With leukopenia n = 717 | | | | Without leukopenia n = 2,404 | | | | P-value[a] (Patients with event) | P-value[b] (Utilization per patient) | P-value[c] (Costs per patient) |
|---|---|---|---|---|---|---|---|---|---|---|---|
| | **Patients with event (% of entire cohort)** | **Mean utilization per patient with resource use (SD), 95% CI** | **Mean cost per patient with resource use (SD) (US$ 2022)** | **Median cost per patient with resource use (IQR) (US$ 2022)** | **Patients with event (% of entire cohort)** | **Mean utilization per patient with resource use (SD), 95% CI** | **Mean cost per patient with resource use (SD) (US$ 2022)** | **Median cost per patient with resource use (IQR) (US$ 2022)** | | | |
| **ED visits** | | | | | | | | | | | |
| All-cause ED visits | 444 (61.9%) | 2.78 (2.83), (2.57–2.98) | 6,409 (13,073) | 2,718 (938–6,282) | 1,291 (53.7%) | 2.53 (2.51), (2.43–2.63) | 5,066 (18,528) | 2,071 (848–5,029) | 0.0001 | 0.0124 | 0.0033 |
| CMV-related ED visits | 45 (6.28%) | 1.27 (0.539), (1.23–1.31) | 3,597 (6,703) | 1,014 (358–2,270) | 30 (1.25%) | 1.2 (0.484), (1.18–1.22) | 2,020 (3,000) | 1,294 (468–2,460) | **<.0001** | 0.5651 | 0.7538 |
| CMV infection | 24 (3.35%) | 1.5 (0.933), (1.43–1.57) | 3,780 (6,272) | 1,713 (835–4,618) | 22 (0.915%) | 1.55 (1.06), (1.5–1.59) | 3,128 (4,010) | 2,154 (429–3,408) | **<.0001** | 0.8601 | 0.8517 |
| csCMVi (ICD-10 codes + treatment change) | 16 (2.23%) | 1.13 (0.342), (1.1–1.15) | 3,626 (6,777) | 721 (254–3108) | 17 (0.707%) | 1.06 (0.243), (1.05–1.07) | 1,439 (1,612) | 941 (383–1,449) | 0.0005 | 0.5388 | 0.9856 |
| CMV disease (ICD-10 codes + treatment change) | 11 (1.53%) | 1.18 (0.405), (1.15–1.21) | 3,067 (4,911) | 788 (358–2,887) | 7 (0.291%) | 1.14 (0.378), (1.13–1.16) | 1,537 (1,615) | 1,449 (193–2,089) | 0.0006 | 0.8886 | 0.6507 |
| Opportunistic infection-related ED visits | 99 (13.8%) | 1.51 (1.24), (1.41–1.6) | 4,966 (10,494) | 1,709 (334–4,441) | 241 (10%) | 1.38 (0.721), (1.35–1.41) | 3,358 (16,862) | 1,325 (383–2,837) | 0.0043 | 0.986 | 0.2271 |
| Toxicity-related ED visits | 117 (16.3%) | 1.41 (0.892), (1.34–1.48) | 3,938 (8,782) | 1,709 (637–3,622) | 56 (2.33%) | 1.18 (0.575), (1.16–1.2) | 2,509 (5,177) | 1,111 (366–2,356) | **<.0001** | 0.0544 | 0.0255 |
| **Dialysis outpatient visits** | | | | | | | | | | | |
| All-cause dialysis visits | 176 (24.5%) | 6.63 (23.6), (4.91–8.36) | 17,263 (126,346) | 656 (258–2,811) | 609 (25.3%) | 3.71 (13), (3.19–4.23) | 4,163 (23,133) | 429 (257–1,253) | 0.6703 | 0.0019 | 0.0158 |
| **Other outpatient visits (not ED or dialysis)** | | | | | | | | | | | |
| All-cause other outpatient visits | 717 (100%) | 56 (22.8), (54.3–57.6) | 55,737 (101,355) | 32,028 (16,185–61,804) | 2404 (100%) | 50 (22.8), (49–50.9) | 45,496 (94,991) | 27,679 (14,877–51,149) | - | **<.0001** | 0.0001 |
| CMV-related other outpatient visits | 228 (31.8%) | 6.86 (7.9), (6.29–7.44) | 8,509 (19,158) | 2,253 (591–7,424) | 305 (12.7%) | 5.64 (9.43), (5.26–6.02) | 4,712 (13,354) | 1,246 (437–3,726) | **<.0001** | 0.0003 | 0.001 |
| CMV infection | 109 (15.2%) | 9.76 (10.5), (8.99–10.5) | 11,443 (25,741) | 3,614 (788–10,905) | 152 (6.32%) | 8.57 (13.2), (8.05–9.1) | 6,691 (16,268) | 1,688 (402–5,966) | **<.0001** | 0.0169 | 0.0104 |
| csCMVi (ICD-10 codes + treatment change) | 61 (8.51%) | 5.25 (5.52), (4.84–5.65) | 5,323 (8575) | 1,524 (500–6,016) | 63 (2.62%) | 4.71 (7.57), (4.41–5.02) | 3,723 (5,994) | 948 (332–5,793) | **<.0001** | 0.1533 | 0.2608 |
| CMV disease (ICD-10 codes + treatment change) | 34 (4.74%) | 6.21 (6.91), (5.7–6.71) | 8,513 (14,415) | 2,163 (197–7479) | 26 (1.08%) | 8.92 (11.1), (8.48–9.37) | 8,068 (16,412) | 1,355 (231–11,031) | **<.0001** | 0.6782 | 0.9465 |

| HCRU at follow-up | With leukopenia n = 717 | | | | Without leukopenia n = 2,404 | | | | P-value[a] (Patients with event) | P-value[b] (Utilization per patient) | P-value[c] (Costs per patient) |
|---|---|---|---|---|---|---|---|---|---|---|---|
| | **Patients with event (% of entire cohort)** | **Mean utilization per patient with resource use (SD), 95% CI** | **Mean cost per patient with resource use (SD) (US$ 2022)** | **Median cost per patient with resource use (IQR) (US$ 2022)** | **Patients with event (% of entire cohort)** | **Mean utilization per patient with resource use (SD), 95% CI** | **Mean cost per patient with resource use (SD) (US$ 2022)** | **Median cost per patient with resource use (IQR) (US$ 2022)** | | | |
| Opportunistic infection-related other outpatient visits | 304 (42.4%) | 6.33 (9.53), (5.63–7.03) | 8859 (35559) | 1513 (362–5986) | 728 (30.3%) | 5.43 (9.61), (5.05–5.82) | 6975 (33188) | 1,141 (367–4,104) | **<.0001** | 0.2349 | 0.2016 |
| Toxicity-related other outpatient visits | 649 (90.5%) | 3.8 (4.04), (3.51–4.1) | 4,319 (8,135) | 1,615 (581–4,545) | 296 (12.3%) | 3.29 (4.18), (3.13–3.46) | 3,761 (9,993) | 967 (387–2,920) | **<.0001** | 0.0004 | 0.0002 |
| **Inpatient hospitalizations** | | | | | | | | | | | |
| All-cause hospitalization visits | 372 (51.9%) | 2.09 (1.57), (1.97–2.2) | 67,373 (100,403) | 38,388 (15,945–78,971) | 937 (39%) | 1.66 (1.26), (1.61–1.71) | 66,558 (163,222) | 25,937 (13,037–61,319) | **<.0001** | **<.0001** | 0.0002 |
| CMV related hospitalization visits | 89 (12.4%) | 1.39 (0.717), (1.34–1.45) | 40,799 (51,221) | 23,825 (11,434–40,478) | 74 (3.08%) | 1.42 (0.993), (1.38–1.46) | 60,553 (109,926) | 27,418 (14,752–59,491) | **<.0001** | 0.5667 | 0.1965 |
| CMV infection | 26 (3.63%) | 1.62 (0.941), (1.55–1.68) | 38,566 (27,515) | 34,151 (12,150–60,962) | 32 (1.33%) | 1.34 (0.602), (1.32–1.37) | 47,881 (50,133) | 29,542 (13,837–58,144) | **<.0001** | 0.3211 | 0.7486 |
| csCMVi (ICD-10 codes + treatment change) | 31 (4.32%) | 1.19 (0.402), (1.16–1.22) | - | - | 21 (0.874%) | 1.24 (0.436), (1.22–1.26) | - | - | **<.0001** | 0.7121 | - |
| CMV disease (ICD-10 codes + treatment change) | 21 (2.93%) | 1.29 (0.561), (1.24–1.33) | 46,121 (60,389) | 17,671 (10,465–77,232) | 11 (0.458%) | 1.91 (1.58), (1.85–1.97) | 83,742 (94,203) | 27,885 (23,867–115,497) | **<.0001** | 0.3154 | 0.1218 |
| Opportunistic infection - related hospitalization visits | 147 (20.5%) | 1.45 (0.93), (1.38–1.52) | 60,814 (109,933) | 30,183 (17,133–61,701) | 287 (11.9%) | 1.4 (0.829), (1.36–1.43) | 72,762 (187,558) | 25,203 (12,356–59,158) | **<.0001** | 0.2507 | 0.0446 |
| Toxicity-related hospitalization visits | 163 (22.7%) | 1.3 (0.659), (1.25–1.35) | 47458 (100550) | 22235 (13474–47884) | 87 (3.62%) | 1.16 (0.37), (1.15–1.18) | 33528 (34741) | 21,122 (11,915–41,010) | **<.0001** | 0.1269 | 0.3091 |
| **All-cause length of stay (days)** | | | | | | | | | | | |
| Total number of hospitalization days | - | 13.2 (15.2), (12.1–14.4) | - | - | - | 10.6 (18.8), (9.84–11.3) | - | - | - | **<.0001** | - |
| Length of stay by visit (days) | - | 5.99 (4.37), (5.54–6.43) | - | - | - | 5.67 (4.77), (5.36–5.98) | - | - | - | 0.0135 | - |
| **Medication counts (inpatient or outpatient)** | | | | | | | | | | | |

| HCRU at follow-up | With leukopenia n = 717 | | | | Without leukopenia n = 2,404 | | | | P-value[a] (Patients with event) | P-value[b] (Utilization per patient) | P-value[c] (Costs per patient) |
|---|---|---|---|---|---|---|---|---|---|---|---|
| | **Patients with event (% of entire cohort)** | **Mean utilization per patient with resource use (SD), 95% CI** | **Mean cost per patient with resource use (SD) (US$ 2022)** | **Median cost per patient with resource use (IQR) (US$ 2022)** | **Patients with event (% of entire cohort)** | **Mean utilization per patient with resource use (SD), 95% CI** | **Mean cost per patient with resource use (SD) (US$ 2022)** | **Median cost per patient with resource use (IQR) (US$ 2022)** | | | |
| CMV-related medical counts | 48 (6.69%) | 4.35 (3.12), (4.13–4.58) | 6,455 (11,979) | 2,224 (688–6,226) | 46 (1.91%) | 6.83 (7.58), (6.52–7.13) | 7,502 (9,957) | 3,277 (1,260–8,487) | **<.0001** | 0.4278 | 0.1932 |
| Toxicity-related medical counts | 308 (43%) | 5.28 (7.96), (4.7–5.86) | 41,502 (103,219) | 2,273 (498–30,121) | 636 (26.5%) | 5.06 (10.6), (4.63–5.48) | 51,213 (118,474) | 2,277 (43–38,699) | **<.0001** | 0.0055 | 0.5181 |
| G-CSF-related medical counts | 172 (24%) | 3.51 (3.86), (3.23–3.79) | 15,206 (50,786) | 1,762 (577–6,061) | 187 (7.78%) | 3.37 (3.89), (3.21–3.52) | 19,064 (56,176) | 1,892 (429–12,411) | **<.0001** | 0.8865 | 0.6074 |
| Blood transfusion-related medical counts | 152 (21.2%) | 3.84 (7.76), (3.27–4.41) | 49,452 (92,288) | 8,621 (0–59,947) | 439 (18.3%) | 3.45 (6.67), (3.18–3.72) | 58,325 (109,930) | 1,285 (0–58,697) | 0.078 | 0.1741 | 0.9115 |
| **Prescription pharmacy counts (NDC)** | | | | | | | | | | | |
| All-cause pharmacy prescriptions | 717 (100%) | 84.9 (34.7), (82.3–87.4) | 32,887 (36,858) | 25,706 (14,598–42,662) | 2,404 (100%) | 84.7 (33.8), (83.4–86.1) | 31,167 (40,477) | 24,009 (15,031–37,076) | - | 0.9453 | 0.0472 |
| CMV-related pharmacy prescriptions | 717 (100%) | 4.83 (2.67), (4.63–5.02) | 11,606 (11,944) | 7,497 (3,665–14,608) | 2,404 (100%) | 4.49 (2.45), (4.39–4.58) | 10,344 (9,491) | 7,544 (4,153–13,653) | - | 0.0042 | 0.6979 |
| Toxicity (G-CSF)-related pharmacy prescriptions | 66 (9.21%) | 2.18 (2.13), (2.03–2.34) | 3,613 (4,519) | 2,132 (903–4,507) | 101 (4.2%) | 1.81 (2.37), (1.72–1.91) | 4,266 (6,418) | 2,354 (1,216–4,525) | **<.0001** | 0.0827 | 0.4624 |
| **Total** | | | | | | | | | | | |
| Total costs | - | - | 131,785 (191,613) | 89,019 (49,525–153,893) | - | - | 106,379 (162,066) | 69,438 (43,438–113,620) | - | - | **<.0001** |

Abbreviations: CI, confidence interval; CMV, cytomegalovirus; csCMVi, clinically significant cytomegalovirus infection; ED, emergency department; G-CSF, granulocyte-colony stimulating factor; HCRU, healthcare resource utilization; ICD, international classification of disease; IQR, interquartile range; NDC, national drug code; SD, standard deviation.

[a] Chi-squared P-value reported for comparison of patients with event (% of entire cohort).

[b] Wilcoxon rank-sum P-value reported for comparison of mean utilization per patient (among those with resource use).

[c] Wilcoxon rank-sum P-value reported for comparison of cost per patient (among those with resource use).

**Table S7. Differences in Adjusted Costs Between Those with and without Neutropenia and with and without Leukopenia, 1 Year Post-Transplant, from Multivariable Models for Clinical Outcomes of Interest[a]**

| Event | Difference (with and without events) ($) | Confidence level low-high ($) | Number of events | Cost difference per event ($) | Difference (with and without events) ($) | Confidence level low-high ($) | Number of events | Cost difference per event ($) | Difference between cohorts ($) |
|---|---|---|---|---|---|---|---|---|---|
| | With neutropenia | | | | Without neutropenia | | | | |
| CMV infection | 18,246 | 14800-22495 | 1.43 | 12,772 | 15,082 | 10,203–14,392 | 1.34 | 11,280 | 3,164 |
| CsCMVi | 57,094 | 45483-71669 | 1.03 | 55,439 | 20,084 | 13093-17373 | 1.04 | 19,302 | 37,010 |
| CMV disease | 62,427 | 46681-83485 | 1.24[b] | 50,473 | 44,958 | 16284-24770 | 1.06 | 42,389 | 17,469 |
| Acute Graft Rejection | 71,329 | 60694-83828 | 1.33 | 53,497 | 36,760 | 32978-61289 | 1.29 | 28,586 | 34,569 |
| Graft Failure | 62,291 | 50744-76465 | 1 | 62,291 | 31,751 | 34305-39391 | 1.00 | 31,751 | 30,540 |
| Viral, fungal, or bacterial opportunistic infection | 34,875 | 30569-39787 | 6.24 | 5,593 | 27,227 | 28519-35349 | 4.86 | 5,598 | 7,648 |
| G-CSF | 12,365 | 10827-14121 | 3.09 | 3,998 | 50,581 | 25587-28972 | 3.18 | 15,924 | -38,216 |
| Re-hospitalization | 79,666 | 70904-89511 | 1.87 | 42,663 | 67,501 | 45514-56212 | 1.59 | 42,392 | 12,165 |
| Other | -56,640 | - | - | - | -41,539 | - | - | - | -15,101 |
| | With leukopenia | | | | Without leukopenia | | | | |
| CMV infection | 17487 | 14477-21123 | 1.40 | 12475 | 13572 | 11676-15776 | 1.34 | 10099 | 3915 |
| CsCMVi | 34104 | 27286-42626 | 1.03 | 33182 | 34640 | 27933-42958 | 1.04 | 33197 | -536 |
| CMV disease | 49155 | 36771-65710 | 1.18[b] | 41834 | 55890 | 40949-76282 | 1.13 | 49503 | -6735 |
| Acute Graft Rejection | 60571 | 52754-69547 | 1.29 | 46921 | 35320 | 32885-37935 | 1.29 | 27297 | 25251 |
| Graft Failure | 61831 | 51664-73998 | 1.00 | 61831 | 27955 | 24980-31284 | 1.00 | 27955 | 33876 |
| Viral, fungal, or bacterial opportunistic infection | 34467 | 30638-38774 | 5.78 | 5958 | 26394 | 24767-28128 | 4.96 | 5326 | 8073 |
| G-CSF | 30683 | 26986-34886 | 3.28 | 9347 | 34597 | 31397-38123 | 3.00 | 11518 | -3914 |
| Re-hospitalization | 75699 | 68157-84076 | 1.91 | 39580 | 67449 | 63782-71327 | 1.56 | 43303 | 8250 |
| Other | -51469 | - | - | - | -41459 | - | - | - | -10010 |

Abbreviations: CMV, cytomegalovirus; csCMVi, clinically significant cytomegalovirus infection; G-CSF, granulocyte-colony stimulating factor.

[a] Analysis excludes patients who died prior to 1-year post-transplant (i.e., died mid-year).

[b] Clinical experts have noted that the CMV disease event rate observed in clinical practice is higher. This elevated rate observed in the database analysis could be attributed to multiple inputs and the misclassification of ICD codes related to CMV. Consequently, it is assumed that the event rate would be 1, signifying one CMV disease event per patient per year.

## Table S8. Unadjusted Costs by Clinical Outcome Event 2-5 Years Post-Transplant[a]

| Event | Patients with neutropenia or leukopenia | | | | Patients without neutropenia or leukopenia | | | |
|---|---|---|---|---|---|---|---|---|
| | Number of patients with ≥1 event (%) | Total Number of events (N) | Cost per. Patient Mean (SD) | Cost per. Patient Median (IQR) | Number of patients with ≥1 event (%) | Total Number of events (N) | Cost per. Patient Mean (SD) | Cost per. Patient Median (IQR) |
| Viral, fungal, or bacterial opportunistic infection | 119 (44.4%) | 658 | $93,144 ($110,529) | $54,496 ($25,398–$108,057) | 1140 (29.8%) | 3661 | $70,493 ($123,360) | $37,595 ($19,217–$71,962) |
| CMV infection | 48 (17.9%) | 79 | $100,977 ($145,142) | $46,943 ($20,037–$81,576) | 209 (5.46%) | 355 | $60,961 ($79,711) | $32,174 ($16,128–$67,422) |
| csCMVi | 6 (2.24%) | 8 | $192,209 ($175,278) | $152,806 ($59,605–$236,356) | 11 (0.287%) | 11 | $119,860 ($89,096) | $125,048 ($55,647–$156,780) |
| CMV disease | 3 (1.12%) | 3 | $250,684 ($233,828) | $151,913 ($82,449–$517,691) | 1 (0.0261%) | 2 | $59,446 (NA) | $59,446 ($59,446–$59,446) |
| G-CSF | 37 (13.8%) | 137 | $141,211 ($245,244) | $64,464 ($43,529–$167,635) | 71 (1.85%) | 274 | $158,487 ($155,326) | $97,868 ($51,887–$211,790) |
| Graft failure | 40 (14.9%) | 40 | $132,402 ($157,505) | $71,090 ($37,582–$159,540) | 440 (11.5%) | 440 | $104,868 ($215,499) | $46,000 ($20,730–$94,911) |
| Acute rejection | 37 (13.8%) | 45 | $174,561 ($257,980) | $74,441 ($42,794–$216,563) | 265 (6.92%) | 329 | $116,669 ($202,737) | $55,056 ($25,169–$117,720) |
| NODAT | 12 (4.48%) | 12 | $51,384 ($42,633) | $41,635 ($15,162–$79,224) | 195 (5.09%) | 195 | $50,063 ($112,800) | $22,296 ($15,010–$48,377) |
| All-cause re-hospitalization | 96 (35.8%) | 307 | $138,380 ($137,938) | $85,688 ($45,775–$178,920) | 744 (19.4%) | 2050 | $117,055 ($165,832) | $70,219 ($43,753–$124,401) |
| Other costs | 70 (26.1%) | - | $37,900 ($106,396) | $19,216 ($11,173–$32,191) | 1,864 (48.7%) | - | $26,723 ($47,967) | $18,343 ($10,020–$31,166) |
| All patients | 268 (100%) | - | $80,237 ($139,591) | $38,608 ($17,015–$80,184) | 3,829 (100%) | - | $50,128 ($104,419) | $25,259 ($13,264–$49,348) |

Abbreviations: CMV, cytomegalovirus; csCMVi, clinically significant cytomegalovirus infection; G-CSF, granulocyte-colony stimulating factor; IQR, interquartile range; NA, not available; NODAT, new onset diabetes after transplant; SD, standard deviation.

**Figure S1. Unadjusted Median HCRU Costs Associated with Neutropenia or Leukopenia (A), Neutropenia Alone (B), and Leukopenia Alone (C) After 1-Year Follow-up**

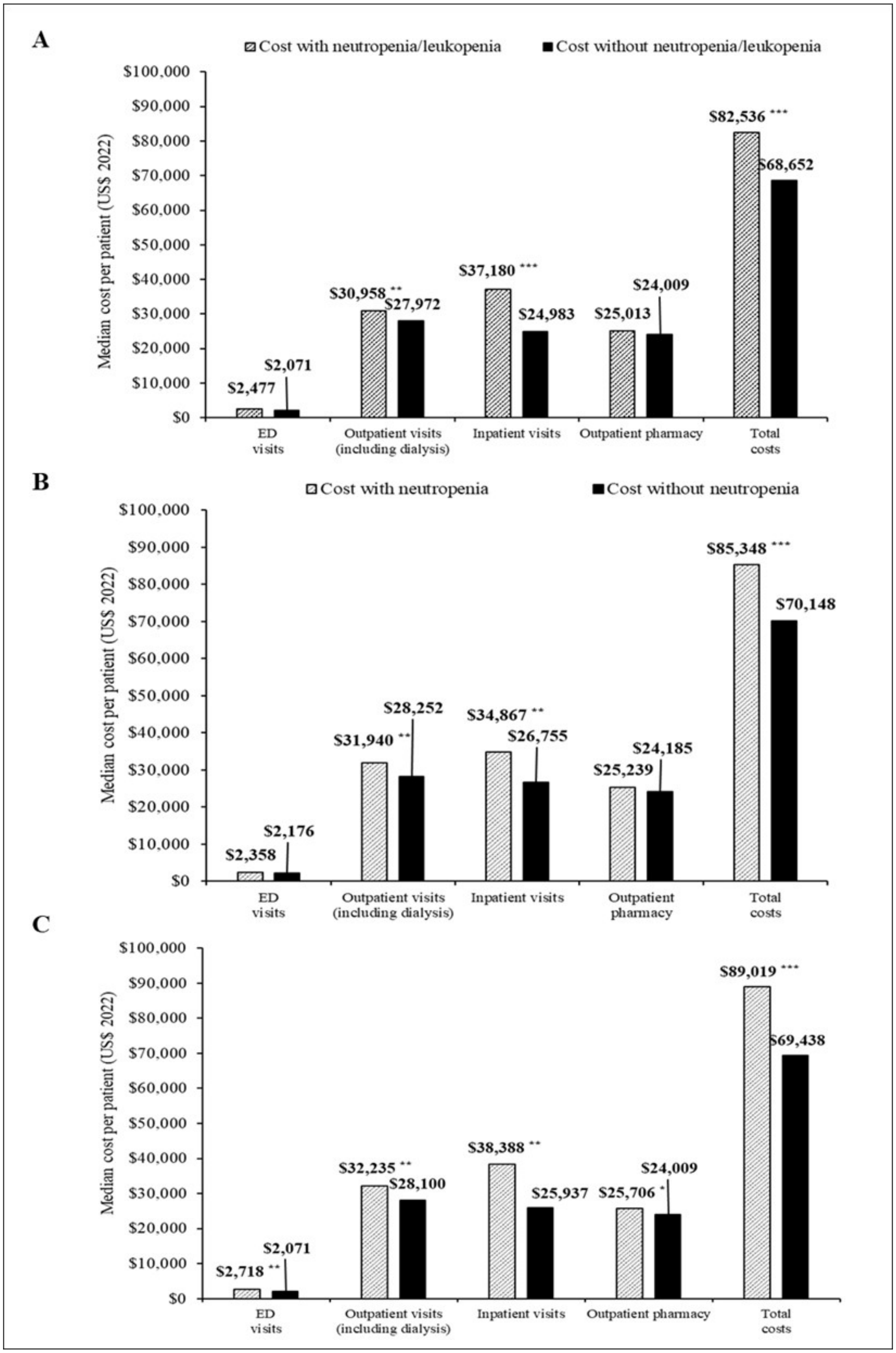


Abbreviations: ED, emergency department; US, United States.

* P<0.05 (Wilcoxon rank-sum; cost per patient using resource).

** P<0.01 (Wilcoxon rank-sum; cost per patient using resource).

*** P<0.0001 (Wilcoxon rank-sum; cost per patient using resource).

**Figure S2. Unadjusted Median HCRU Costs Associated with Neutropenia or Leukopenia (A), Neutropenia Alone (B), and Leukopenia Alone (C) after 2- to 5-Year Follow-up**

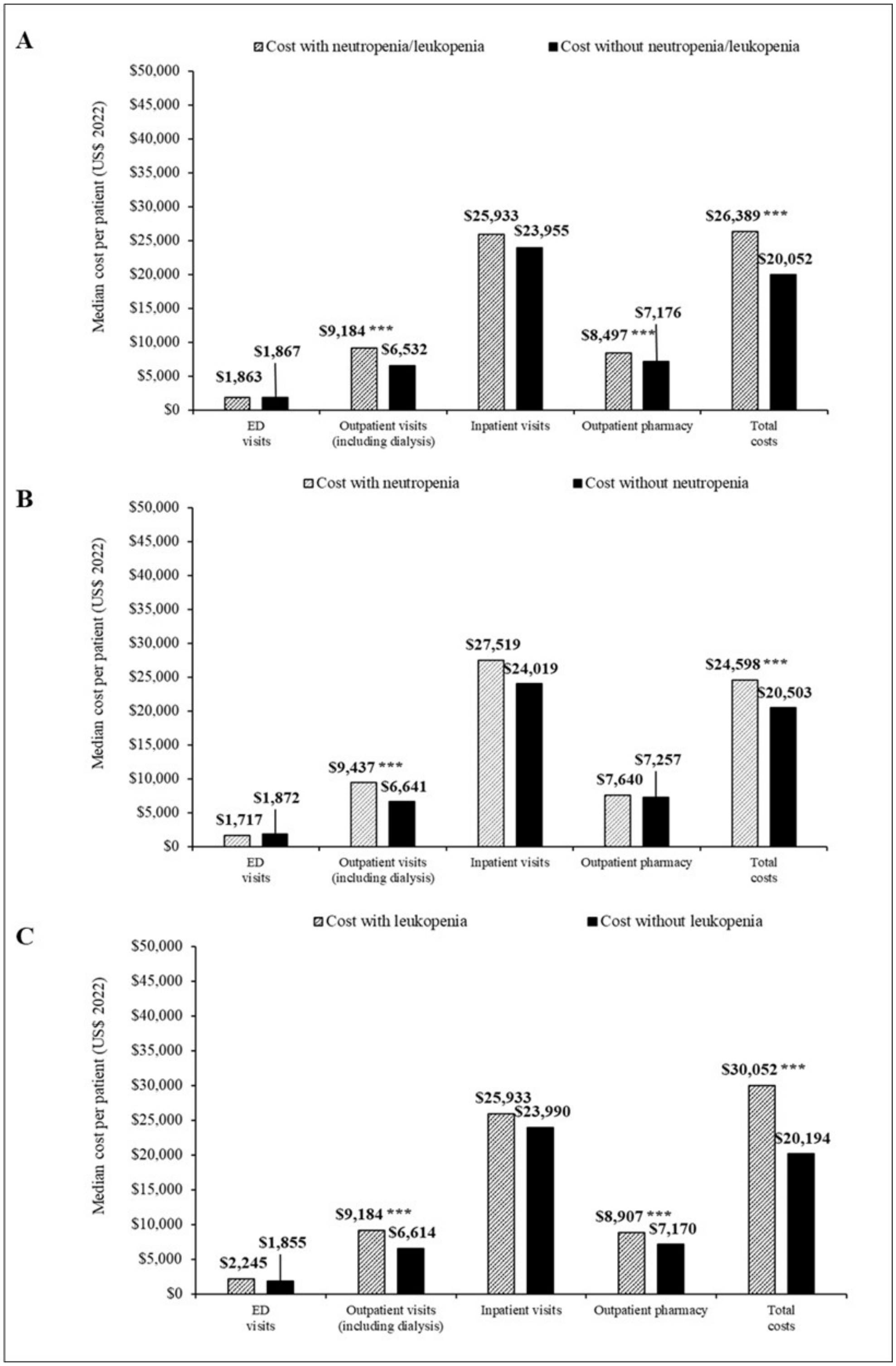


Abbreviations: ED, emergency department; US, United States.

* P<0.05 (Wilcoxon rank-sum; cost per patient using resource).

** P<0.01 (Wilcoxon rank-sum; cost per patient using resource).

*** P<0.0001 (Wilcoxon rank-sum; cost per patient using resource).
